# Supplementary material for: Associations between insomnia and pregnancy and perinatal outcomes: Evidence from mendelian randomization and multivariable regression analyses
Source: PLoS Med. 2022 Sep 6;19(9):e1004090. doi: 10.1371/journal.pmed.1004090 (PMC9488815; doi:10.1371/journal.pmed.1004090)
Supplement: S1 Fig — (DOCX) [file pmed.1004090.s003.docx]

**S1 Fig. Flow chart of each cohort**

1. ***UK Biobank (UKB)***


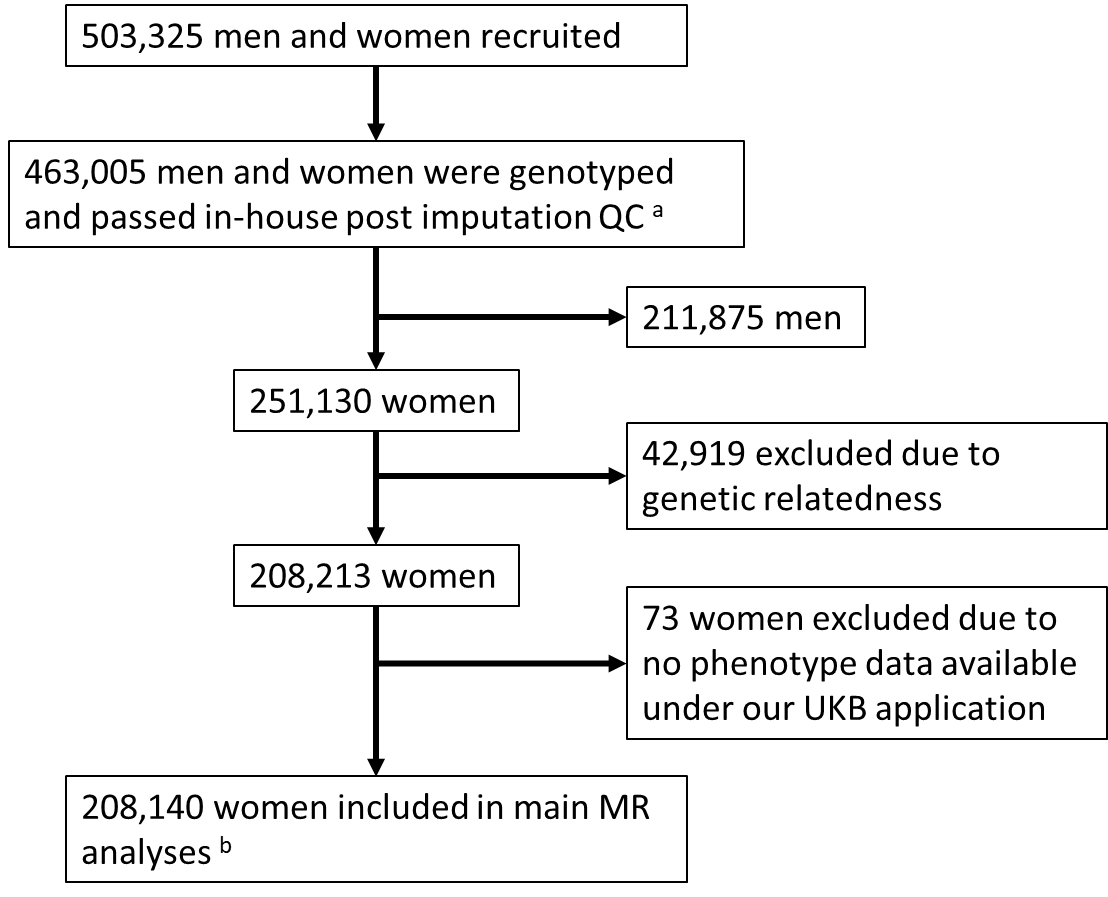


1. ***Avon Longitudinal Study of Parents and Children (ALSPAC)***


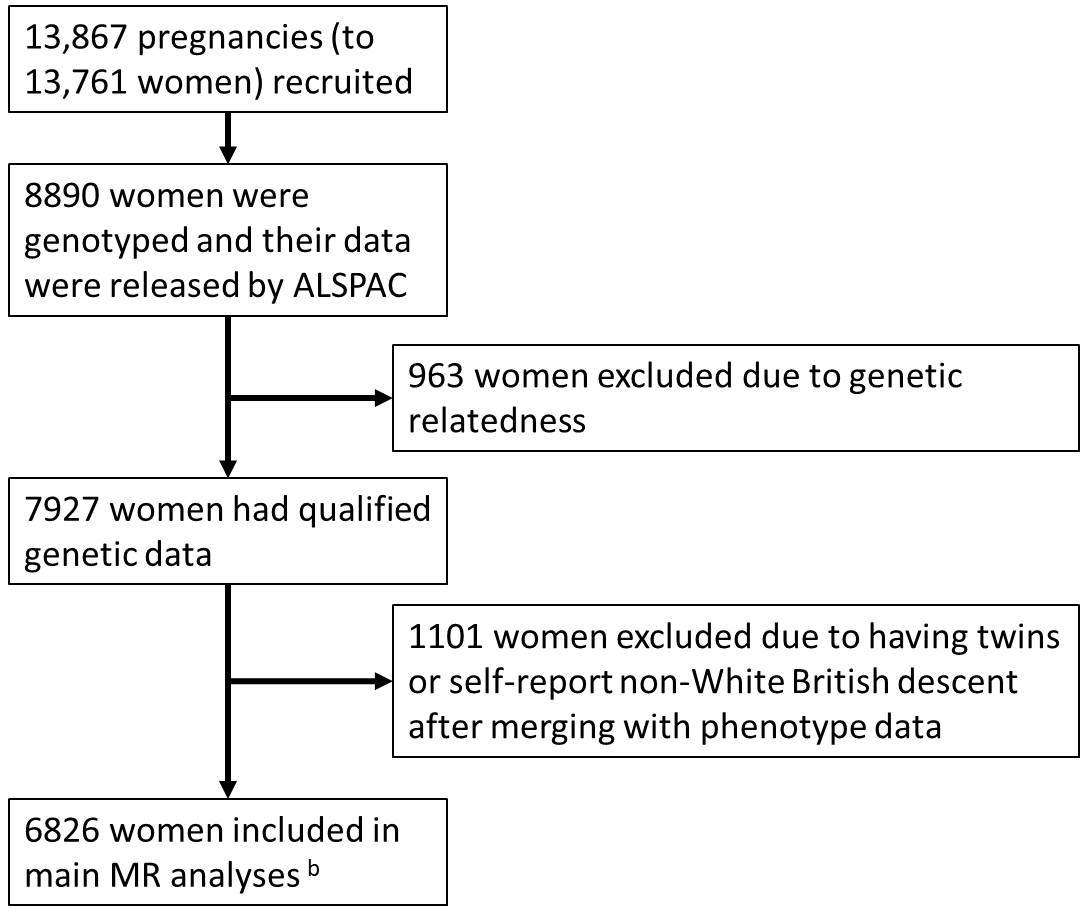


1. ***Born in Bradford (BiB)***


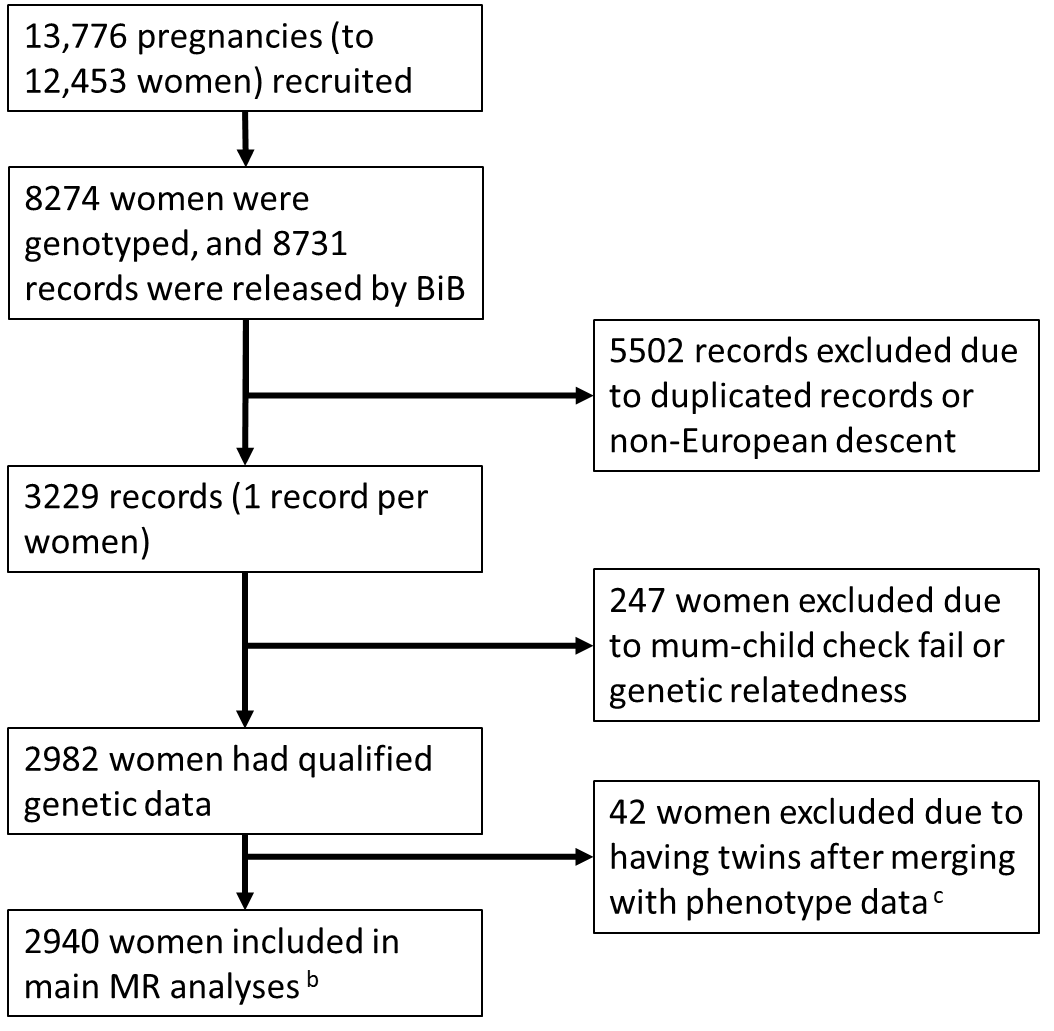


1. ***The Norwegian Mother, Father and Child Cohort Study***


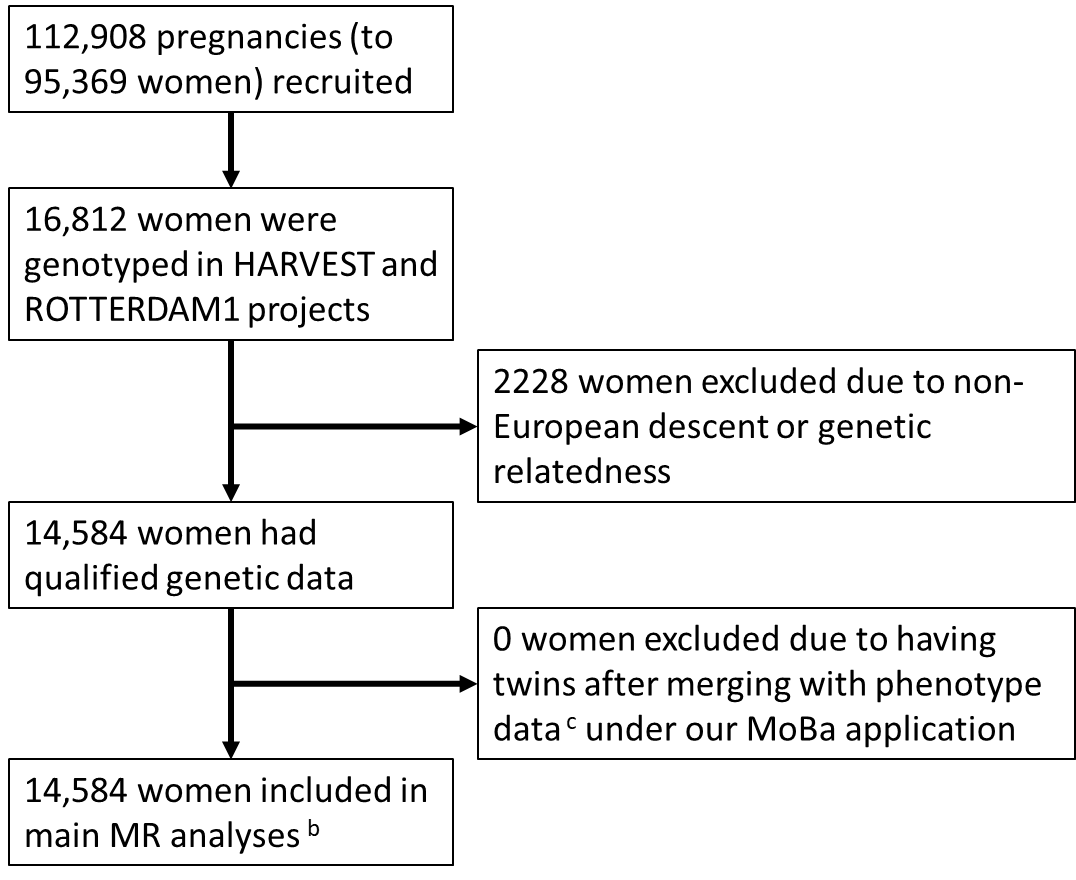


^a^ We used the imputed data released by UKB in March 2018, and applied in-house post-imputation QC [1].

^b^ The numbers of cases and controls for each pregnancy and perinatal outcomes are listed in Table 1.

^c^ We randomly selected one pregnancy per woman in the phenotype data if multiple pregnancies were recorded.

Abbreviation: Mendelian randomization, MR; Quality control, QC.

**References**

1. Mitchell R, Hemani G, Dudding T, Corbin L, Harrison S, Paternoster L. UK Biobank Genetic Data: MRC-IEU Quality Control, version 2. 2019 Jan 22 [cited 1 January 2020]. Available from: <https://data.bris.ac.uk/data/dataset/1ovaau5sxunp2cv8rcy88688v>.
