# Supplementary material for: Associations between insomnia and pregnancy and perinatal outcomes: Evidence from mendelian randomization and multivariable regression analyses
Source: PLoS Med. 2022 Sep 6;19(9):e1004090. doi: 10.1371/journal.pmed.1004090 (PMC9488815; doi:10.1371/journal.pmed.1004090)
Supplement: S2 Fig — (DOCX) [file pmed.1004090.s004.docx]

**S2 Fig. Leave-one (study)-out analyses of MR IVW estimates for causal associations of insomnia with adverse pregnancy and perinatal outcomes across (a) all cohorts and (b) non-UKB cohorts.**

1. all cohorts


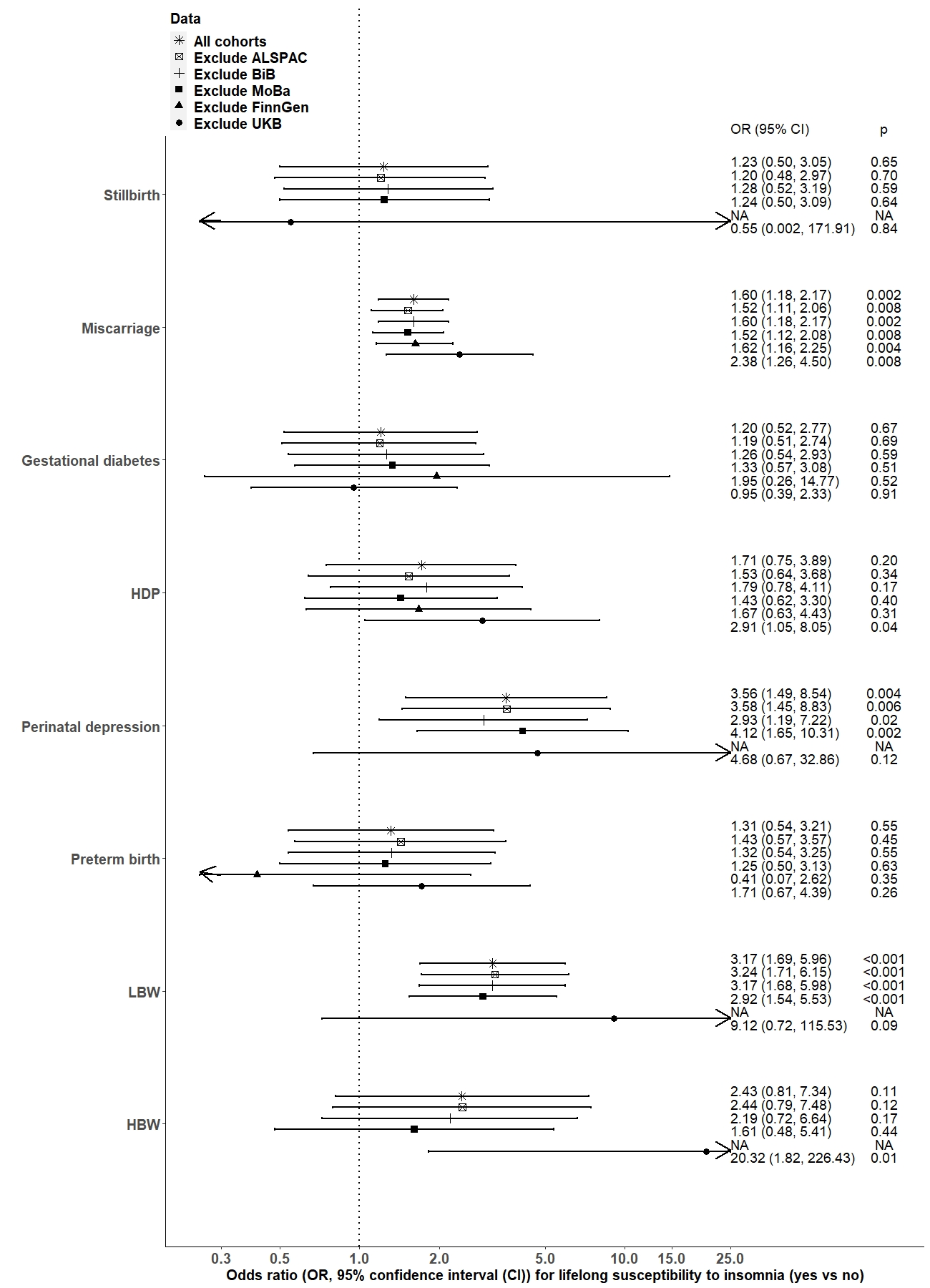


1. non-UKB cohorts


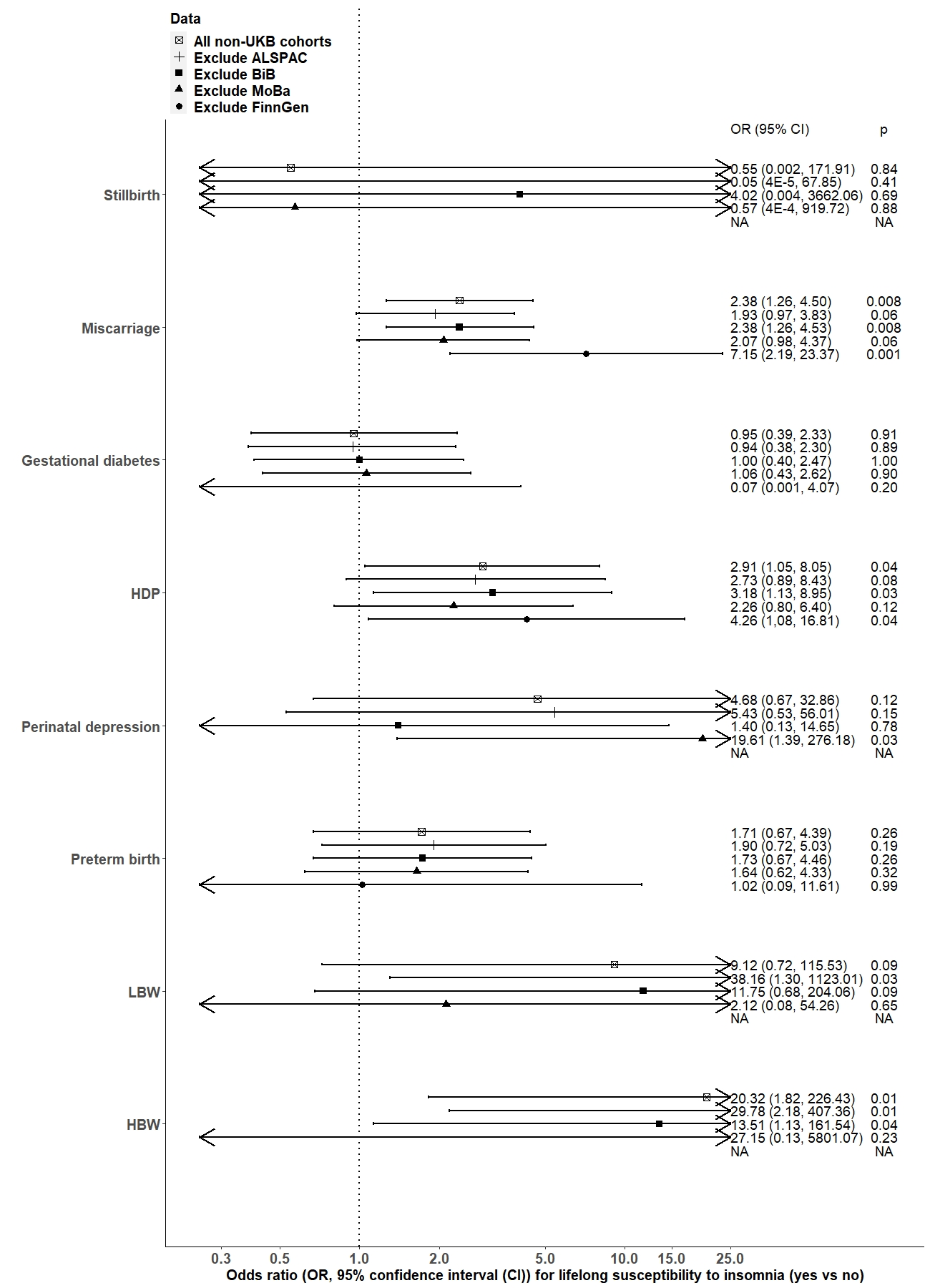


Abbreviations: ALSPAC, Avon Longitudinal Study of Parents and Children; BiB, Born in Bradford; HBW, high offspring birthweight; HDP, hypertensive disorders of pregnancy; IVW, inverse variance weighted; LBW, low offspring birthweight; MR, Mendelian randomization; MoBa, The Norwegian Mother, Father and Child Cohort Study; NA, not applicable; UKB, UK Biobank.
