# Supplementary material for: Associations between insomnia and pregnancy and perinatal outcomes: Evidence from mendelian randomization and multivariable regression analyses
Source: PLoS Med. 2022 Sep 6;19(9):e1004090. doi: 10.1371/journal.pmed.1004090 (PMC9488815; doi:10.1371/journal.pmed.1004090)

**S3 Fig. Leave-one (single nucleotide polymorphisms)-out sensitivity analysis for insomnia on pregnancy and perinatal outcomes in UK Biobank (dataset A on dataset B)**

1. Stillbirth


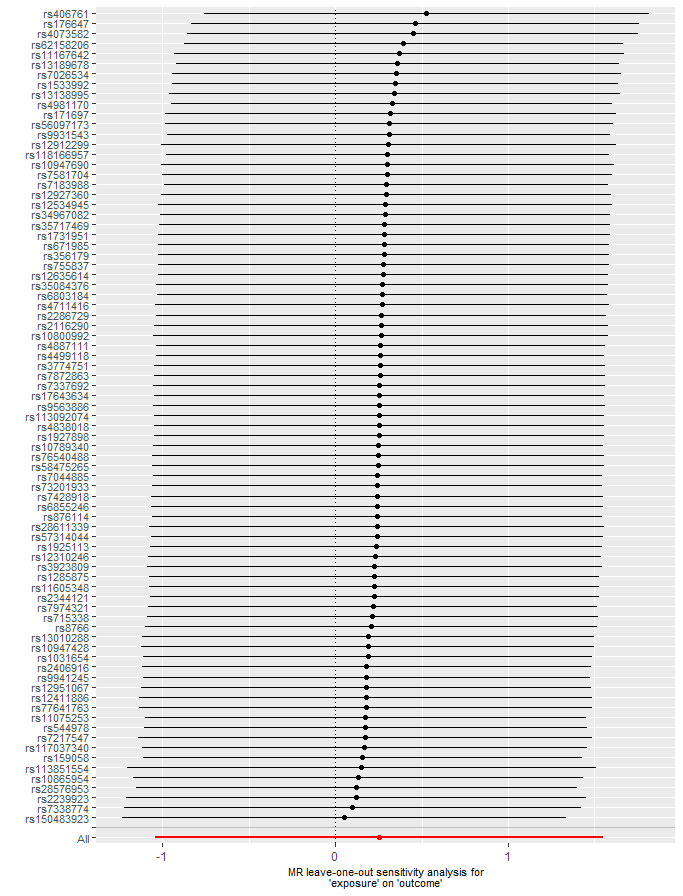


1. Miscarriage


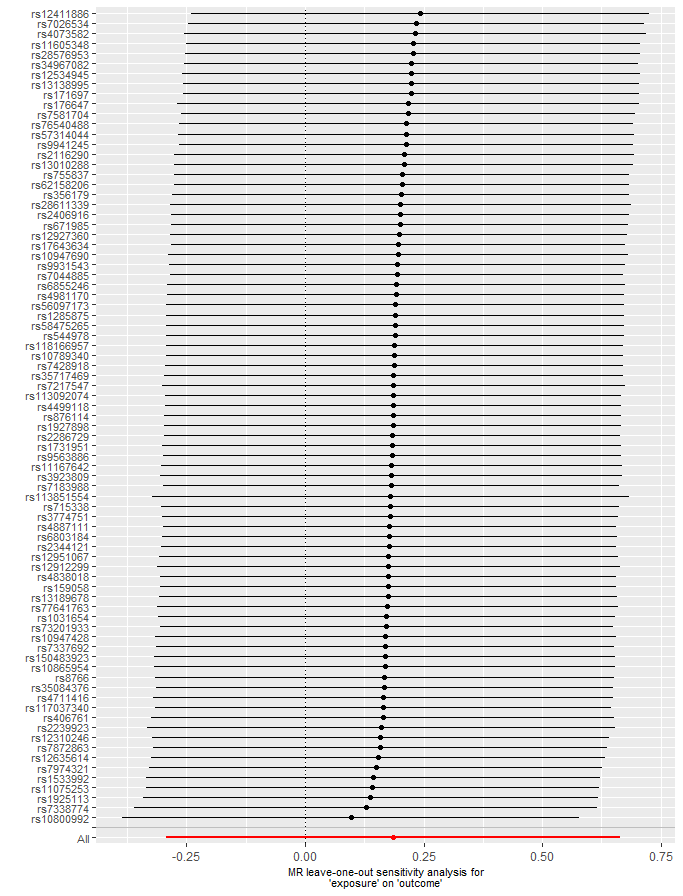


1. Gestational diabetes


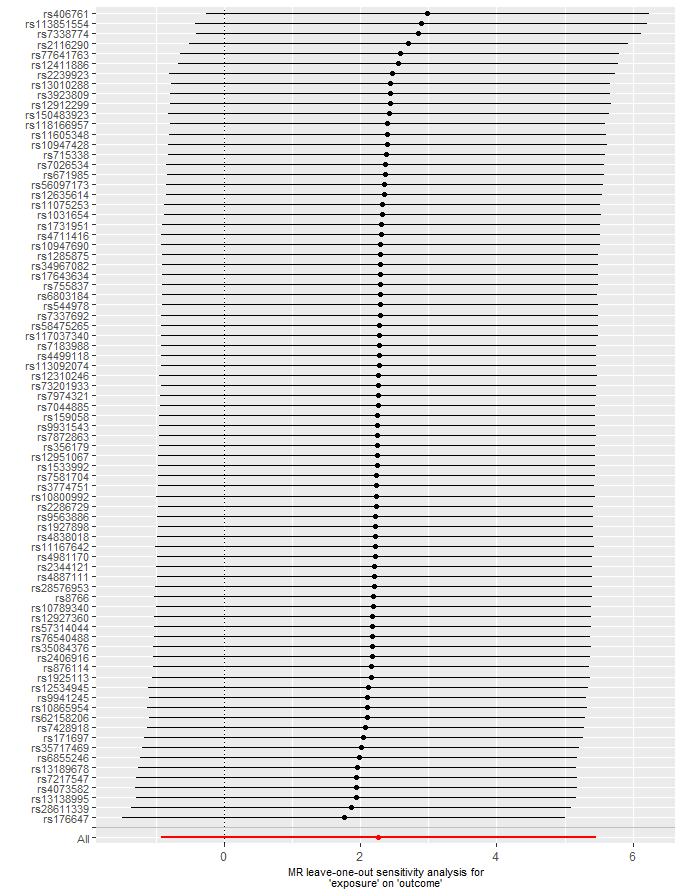


1. Hypertensive disorders of pregnancy


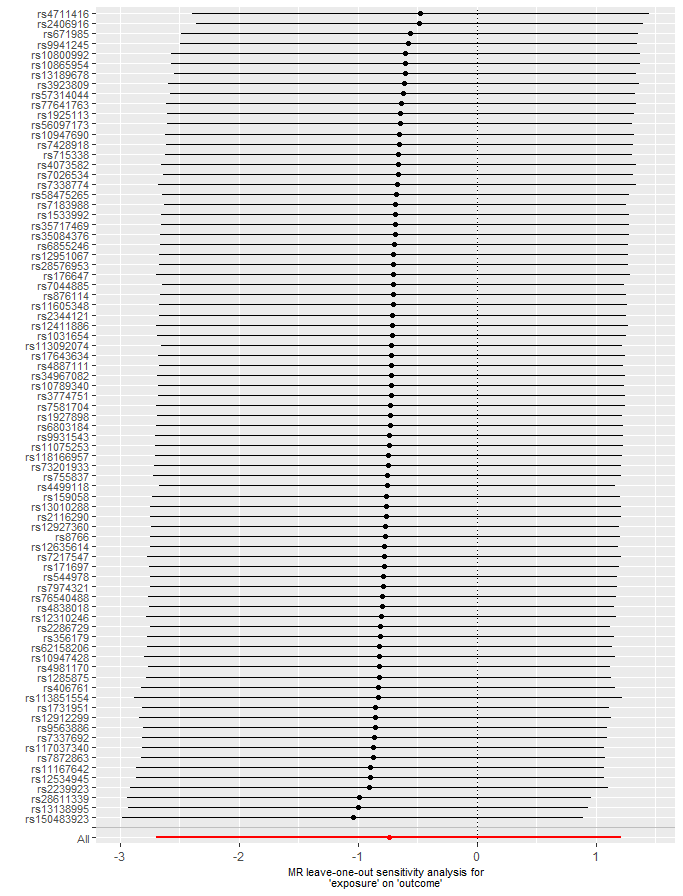


1. Perinatal depression


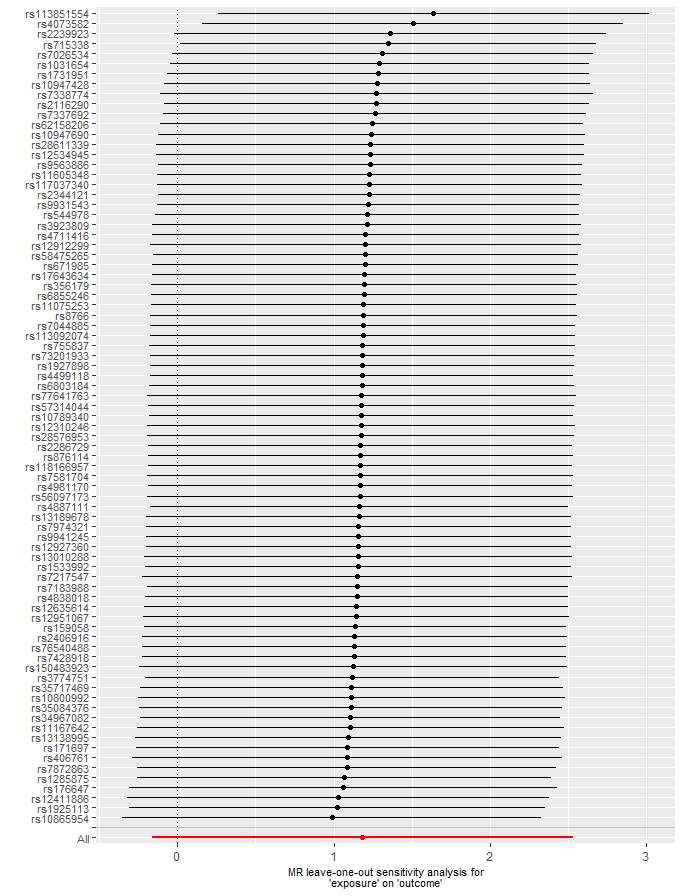


1. Preterm birth


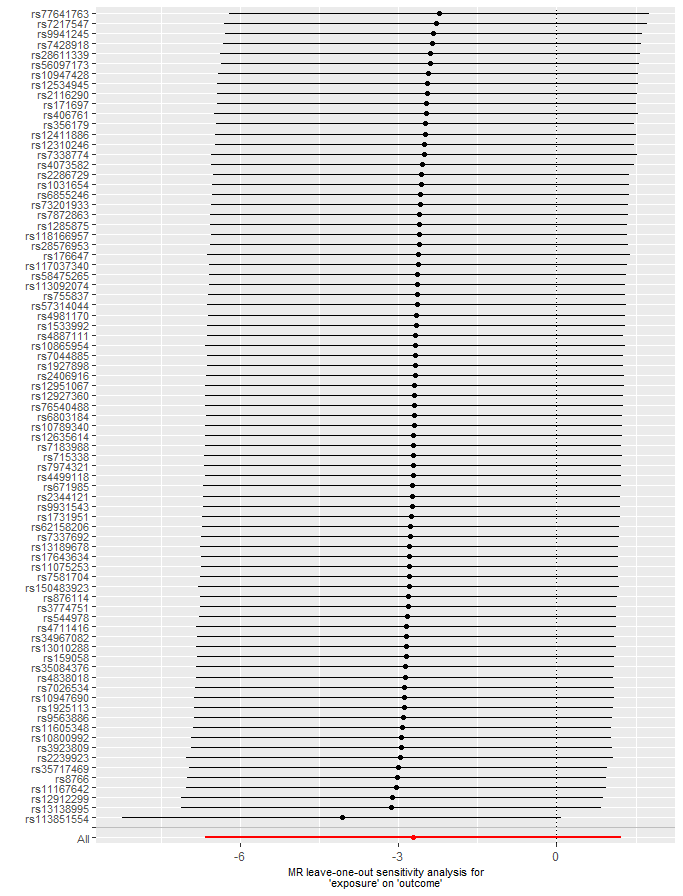


1. Low offspring birthweight


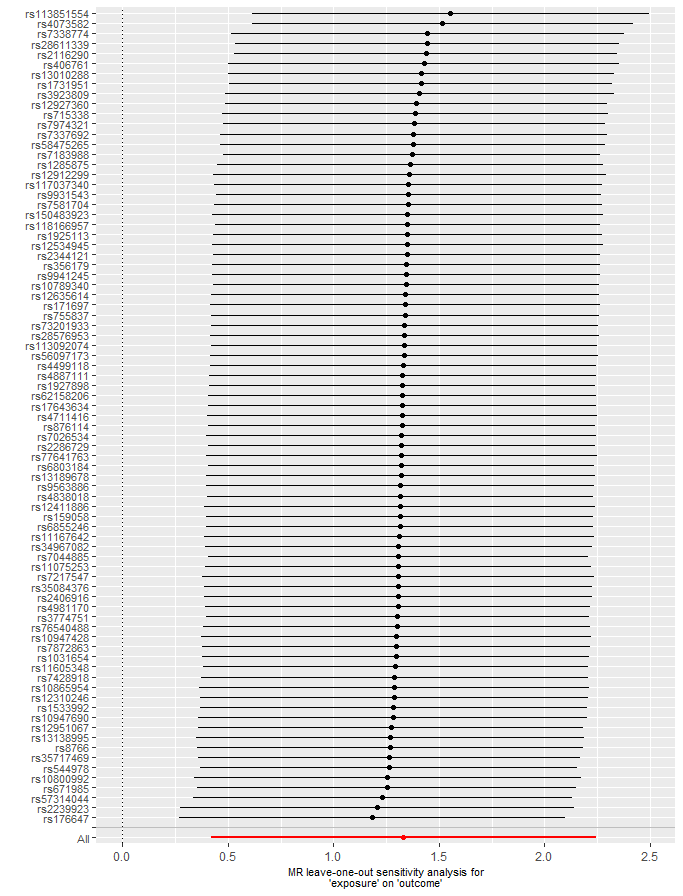


1. High offspring birthweight


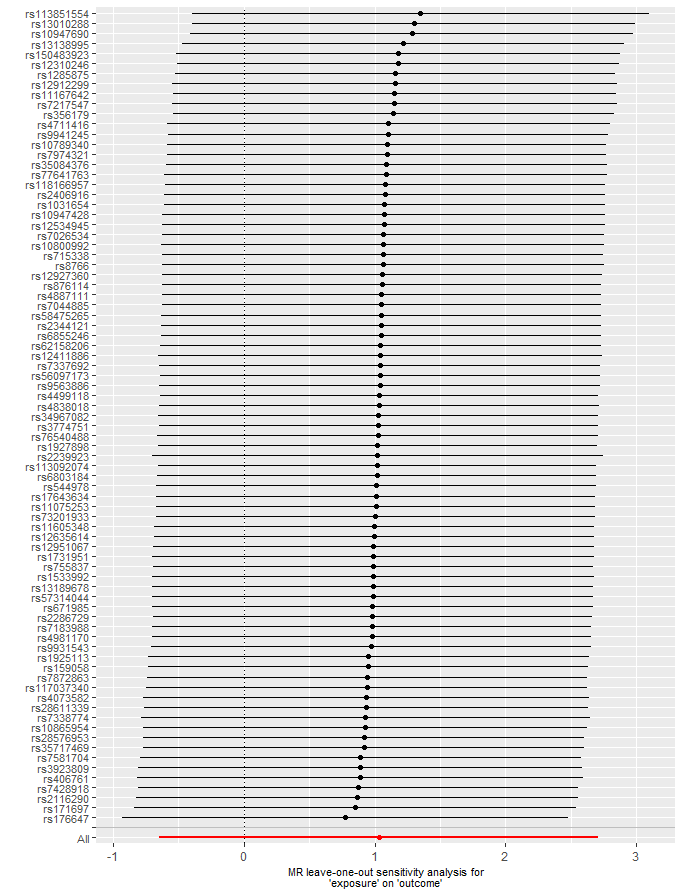

Supplement: S3 Fig — (DOCX) [file pmed.1004090.s005.docx]
