# Supplementary material for: Associations between insomnia and pregnancy and perinatal outcomes: Evidence from mendelian randomization and multivariable regression analyses
Source: PLoS Med. 2022 Sep 6;19(9):e1004090. doi: 10.1371/journal.pmed.1004090 (PMC9488815; doi:10.1371/journal.pmed.1004090)
Supplement: S5 Fig — (DOCX) [file pmed.1004090.s007.docx]

**S5 Fig. Leave-one (single nucleotide polymorphisms)-out sensitivity analysis for insomnia on pregnancy and perinatal outcomes in Avon Longitudinal Study of Parents and Children (ALSPAC), Born in Bradford (BiB), The Norwegian Mother, Father and Child Cohort Study (MoBa), and FinnGen**

1. Stillbirth


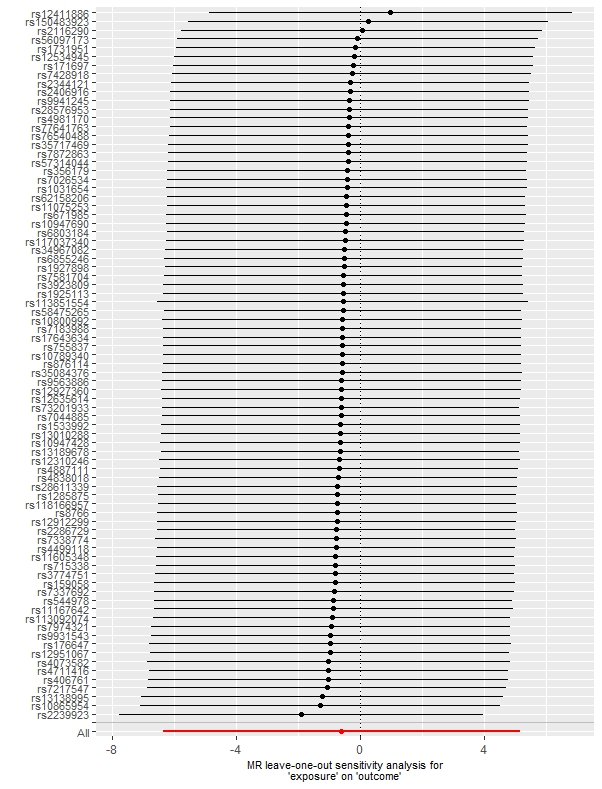


*This analysis only included ALSPAC, BiB and MoBa.

1. Miscarriage


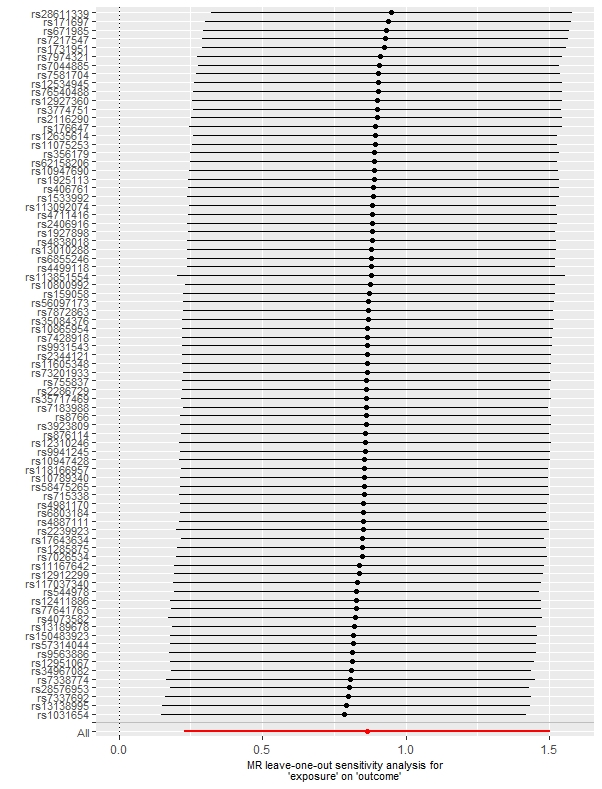


*This analysis included ALSPAC, BiB, MoBa and FinnGen.

1. Gestational diabetes


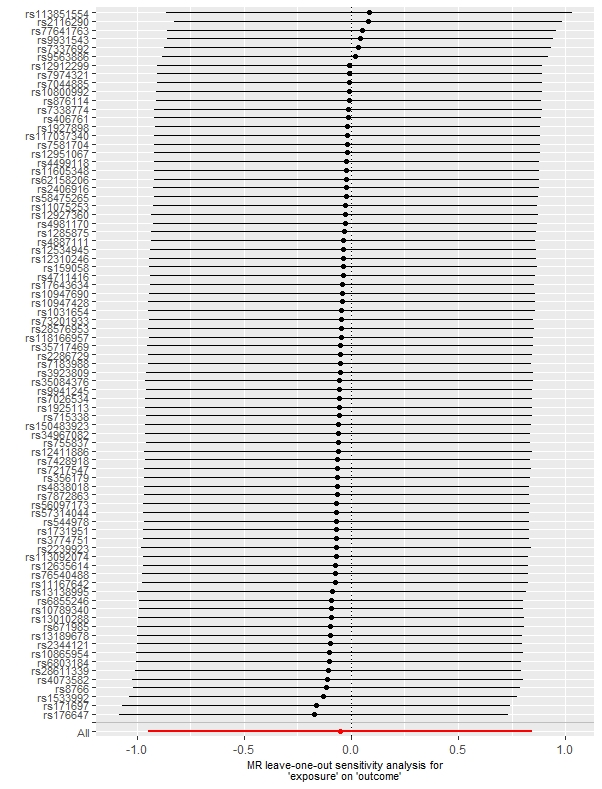


*This analysis included ALSPAC, BiB, MoBa and FinnGen.

1. Hypertensive disorders of pregnancy


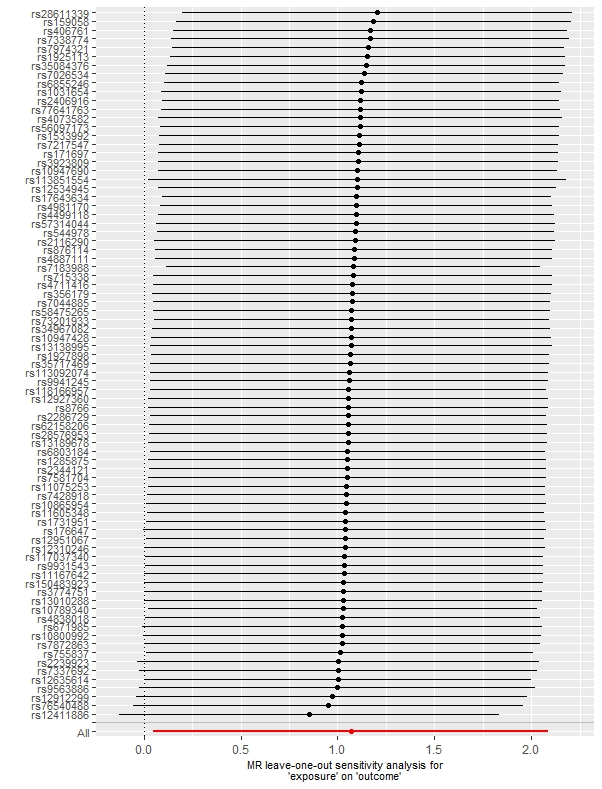


*This analysis included ALSPAC, BiB, MoBa and FinnGen.

1. Perinatal depression


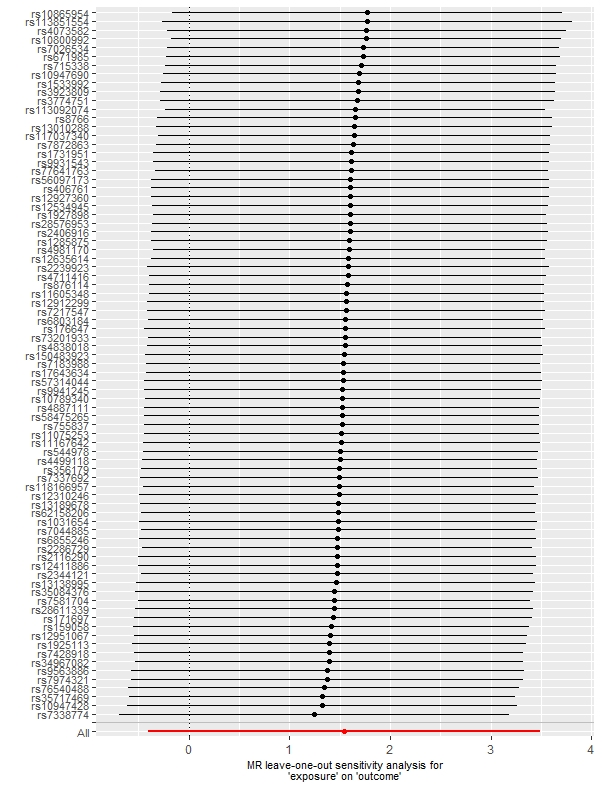


*This analysis only included ALSPAC, BiB and MoBa.

1. Preterm birth


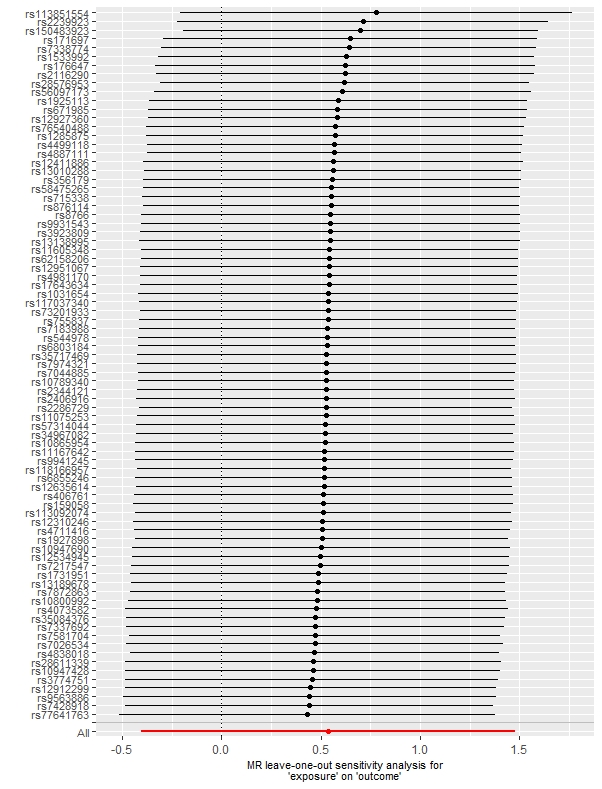


*This analysis included ALSPAC, BiB, MoBa and FinnGen.

1. Low offspring birthweight


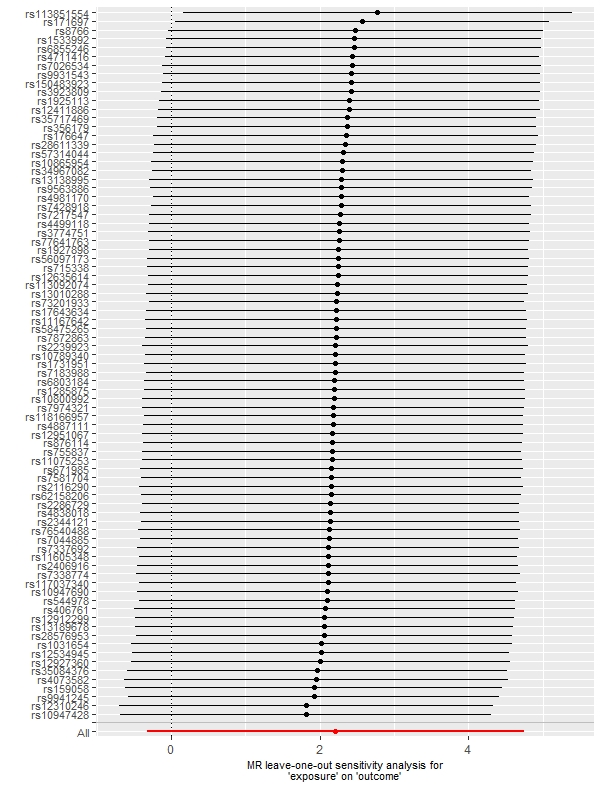


*This analysis only included ALSPAC, BiB and MoBa.

1. High offspring birthweight


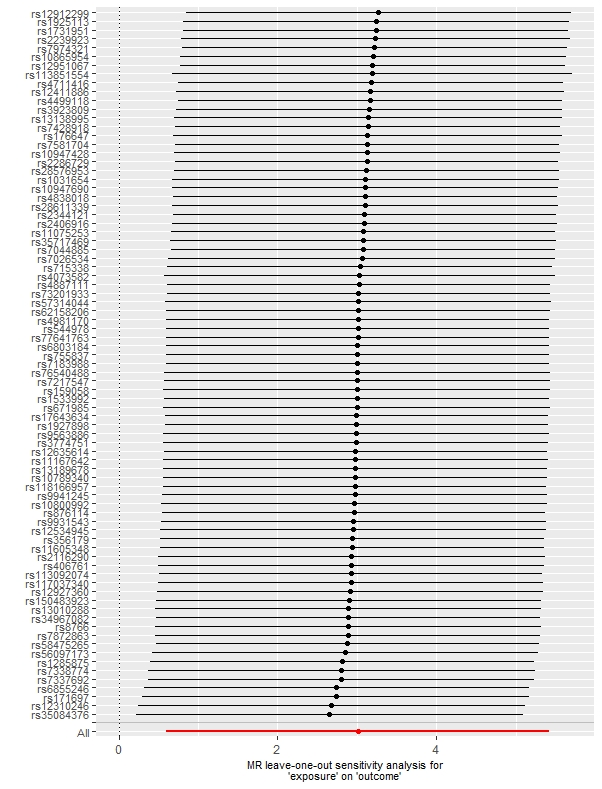


*This analysis only included ALSPAC, BiB and MoBa.
