# Supplementary material for: Associations between insomnia and pregnancy and perinatal outcomes: Evidence from mendelian randomization and multivariable regression analyses
Source: PLoS Med. 2022 Sep 6;19(9):e1004090. doi: 10.1371/journal.pmed.1004090 (PMC9488815; doi:10.1371/journal.pmed.1004090)
Supplement: S6 Fig — (DOCX) [file pmed.1004090.s008.docx]

**S6 Fig. Associations of 81 maternal SNPs with pregnancy and perinatal outcomes comparing unadjusted to adjusted for fetal genotypes (N=18,663 mother-offspring pairs from three birth cohorts)**


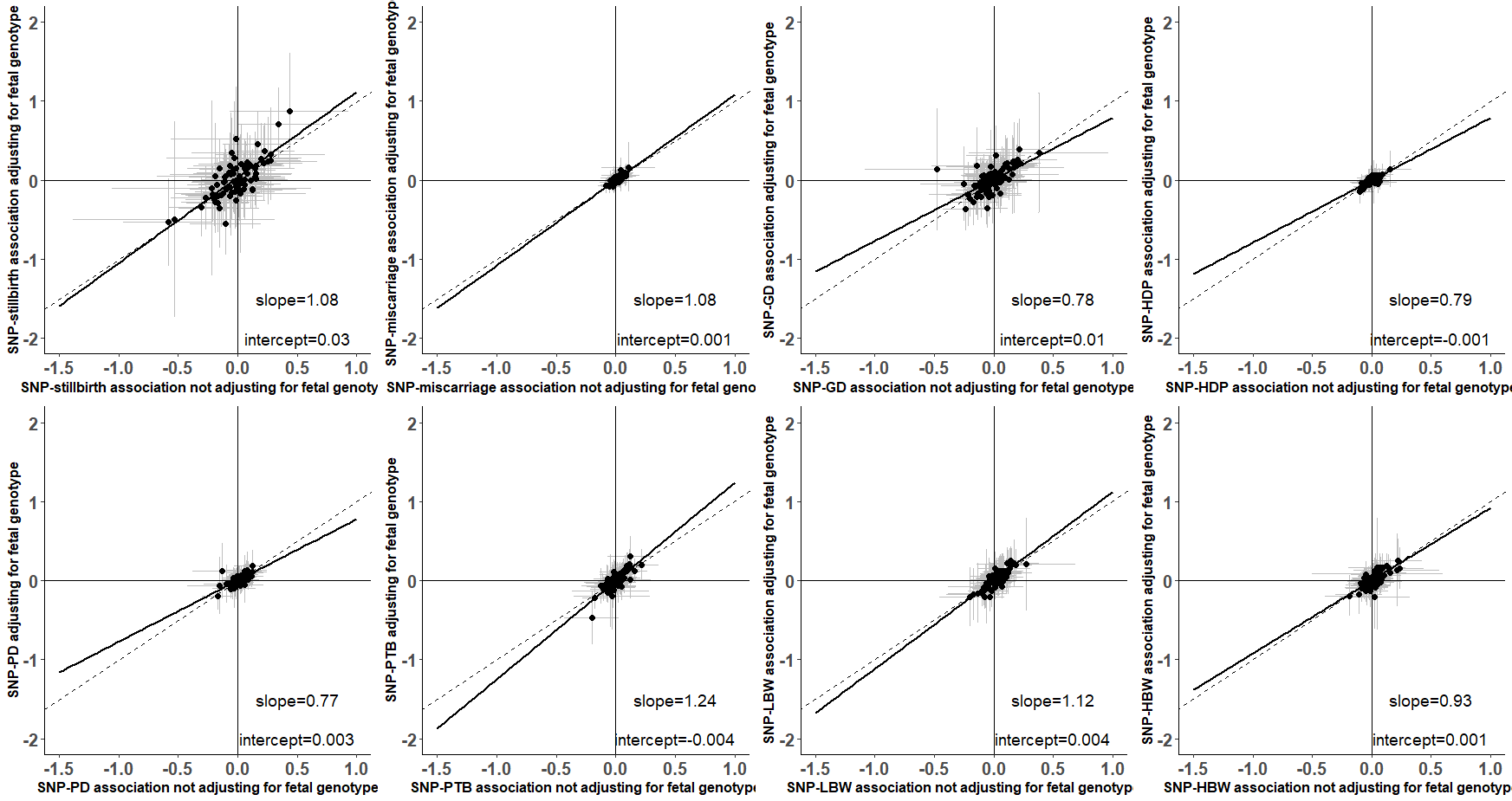


Black dots represent coordinates of ln (odds ratio) without fetal genotype adjustment (x-axis) and with that adjustment (y-axis) and the grey lines represent their 95% confidence intervals. Black solid lines are the fitted linear regression lines through the with and without fetal genotype adjustment ln (odds ratio). The black dash lines represent the line of perfect agreement between the ln (odds ratio) with and without fetal genotype adjustment. We consider a significant alteration of SNP-outcome associations if the slope is ≤0.8 or ≥1.2.

Abbreviations: GD, gestational diabetes; HBW, high offspring birthweight; HDP, hypertensive disorders of pregnancy; LBW, low offspring birthweight; PD, perinatal depression; PTB, preterm birth; SNPs, single nucleotide polymorphisms.
