# Supplementary material for: Associations between insomnia and pregnancy and perinatal outcomes: Evidence from mendelian randomization and multivariable regression analyses
Source: PLoS Med. 2022 Sep 6;19(9):e1004090. doi: 10.1371/journal.pmed.1004090 (PMC9488815; doi:10.1371/journal.pmed.1004090)
Supplement: Attachment — Submitted filename: response to reviewers comments_KT_DAL_submit.docx [file pmed.1004090.s016.docx]

Comments from the Academic Editor:

In the introduction there is some conflation between insomnia and supine sleep position which I do not think is helpful. It seems likely that associations between supine sleep position and perinatal outcomes are mediated by mechanisms distinct from those described as insomnia.

Thank you for pointing this out. We have removed the relevant sentence from introduction.

(page 6, line 90) We deleted: “For example, meta-analyses combining four case-control studies showed that going to sleep in a supine position (plausibly exacerbating sleep-disordered breathing that contributes to decreased sleep quality [1,2]) was associated with higher risk of stillbirth [3] and small-for-gestational age (SGA) [4].

In the ALPSAC logistic regression the numbers of miscarriages and stillbirths seem very high. I may have misunderstood the methodology but I think they have looked at causal effects of reported insomnia at 18 weeks and any previously reported pregnancy loss. I do not think this is correct- and would recommend that they look at the effect of insomnia reported at 18 weeks on any subsequent pregnancy complication in the same pregnancy.

Thank you for the suggestion. We had originally explored associations with ‘any history’ of stillbirth and miscarriage as there are small numbers of miscarriage and stillbirth cases in the index pregnancy in ALSPAC and for miscarriage we were concerned about selection bias (i.e. missing cases that occurred before recruitment. Having considered this further, we feel exploring ‘any history’ for these two outcomes is appropriate for the genetic Mendelian randomisation analyses but not the multivariable regression analyses. We agree that multivariable regression of insomnia at 32 weeks of gestation could be vulnerable to reverse causality for some outcomes. Thus, we have removed multivariable regression results for miscarriage and stillbirth history and for insomnia at 32 weeks of gestation, and revised our manuscript as follows:

***In Abstract,***

(page 2, line 38) From: “Multivariable regression showed associations of insomnia at 18 weeks of gestation with miscarriage (OR 1.30, 95% CI: 1.12, 1.51), stillbirth (OR 2.10, 95% CI: 1.20, 3.69), and perinatal depression (OR 2.96, 95% CI: 2.42, 3.63), but not with LBW (OR 0.92, 95% CI: 0.69, 1.24).”

To: “Multivariable regression showed associations of insomnia at 18 weeks of gestation with perinatal depression (OR 2.96, 95% CI: 2.24, 3.63), but not with LBW (OR 0.92, 95% CI: 0.69, 1.24). Multivariable regression with miscarriage and stillbirth was not possible due to small numbers in index pregnancies.”

***In Author summary,***

(page 4, line 64) From: “To triangulate with our Mendelian randomization estimates, we conducted multivariable regression … measured in pregnancy.”

To: “To triangulate with our MR estimates, we conducted multivariable regression … measured in pregnancy for all outcomes except miscarriage and stillbirth for which there were too few cases in the index pregnancy.”

(page 4, line 68) From: “We found consistent evidence from Mendelian randomization and multivariable regression that insomnia was associated with higher risks miscarriage and perinatal depression, and Mendelian randomization also suggested an unfavourable effect on low offspring birthweight.”

To: “We found consistent evidence from MR and multivariable regression that insomnia was associated with a higher risk of perinatal depression, and MR also suggested unfavourable associations with miscarriage and low offspring birthweight.”

***In Introduction,***

(page 8, line 129) From: “(II) compare those findings with conventional multivariable regression analyses of self-reported insomnia during pregnancy with the same outcomes.”

To: “(II) compare MR findings with conventional multivariable regression analyses of self-reported insomnia during pregnancy with these outcomes, where possible.”

***In Methods,***

(page 10, line 165) From: “However, numbers were too small for reliable results in either MR or multivariable regression, and for miscarriage … prior to recruitment.”

To: “However, numbers were too small for reliable results, and for miscarriage … prior for recruitment. Therefore, we used MR to explore the association of susceptibility to insomnia on a history of miscarriage and stillbirth, and did not undertake any multivariable regression analyses for these two outcomes as suggested during peer review.”

(page 16, line 284) From: “we explored the observational associations of insomnia at 18 and 32 weeks of gestation … adjustment for measured confounders.”

To: “we explored the observational association of insomnia at 18 weeks of gestation … adjustment for measured confounders. During peer review, insomnia at 32 weeks of gestation was not considered in the analysis due to potential reverse causality for some outcomes.”

(page 16, line 287) We deleted: “We had to regress stillbirth history on insomnia at 18 and 32 weeks of gestation, and miscarriage history on insomnia at 18 weeks of gestation, assuming that insomnia in the index pregnancy could also represent the situations in previous pregnancies.”

(page 20, line 341) ***In Results,*** all mention of associations with miscarriage and stillbirth and of insomnia at 32 weeks of gestation from multivariable regression were removed.

***In Discussion,***

(page 23, line 387) From: “Results for miscarriage and perinatal depression are replicated in multivariable regression analyses, but with the exception of results for LBW, which were much closer to the null.”

To: “In multivariable regression, we were unable to assess associations with miscarriage in the index pregnancy. Results for perinatal depression replicated the MR results, but this was not the case for LBW, for which the ORs were much closer to the null.”

(page 24, line 391) From: “Thus, our novel findings of a possible effect of insomnia on miscarriage in both MR and multivariable regression analyses warrants replication.”

To: “Thus, our novel finding of a possible association of insomnia with miscarriage in MR warrants replication, and larger studies that support analyses with both miscarriage and stillbirth would be valuable.”

(page 25, line 422) We deleted: “To our knowledge, this is the first study to explore associations with miscarriage and stillbirth, using either multivariable regression or MR.”

(page 26, line 440) From: “Whilst we cannot test this assumption, the similarity of our MR and multivariable regression estimates for miscarriage and perinatal depression, provides some evidence that it may not have been violated for these outcomes.”

To: “The monotonicity assumption cannot be tested.”

(page 27, line 465) We deleted: “Both MR and multivariable regression analyses of miscarriage and stillbirth assessed the associations of ever experiencing either of them mainly up to the point of recruitment. For the multivariable regression analysis in ALSPAC, insomnia was reported after the outcomes had occurred and it is possible that previous miscarriage or stillbirth might have influenced sleep in the index pregnancy (where insomnia was reported, e.g. due to anxiety [5].”

(page 27, line 472) We added: “Multivariable regression results for maternal outcomes could also be vulnerable to reverse causality, as tendency towards the outcomes might have influenced insomnia reported at 18 weeks of gestation.”

(page 27, line 476) From: “The similarity of the multivariable regression and MR results for miscarriage, stillbirth, GD …”

To: “The similarity of the multivariable regression and MR results for GD …”

(page 14, line 246) ***In Fig 1,***we deleted: “Insomnia at the 32^nd^ week of gestation, N=11,285”

***In Fig 3,*** we removed results for stillbirth and miscarriage, and for insomnia at 32 weeks of gestation.

(page 21, line 352) From: “Fig 3. Multivariable regression associations of insomnia at 18 and 32 weeks of gestation with adverse pregnancy and perinatal outcomes in Avon Longitudinal Study of Parents and Children (ALSPAC).”

To: “Fig 3. Multivariable regression associations of insomnia at 18 weeks of gestation with adverse pregnancy and perinatal outcomes in Avon Longitudinal Study of Parents and Children (ALSPAC).”

(page 22, line 358) We deleted: “^c^ We only report results for insomnia at 18 weeks for miscarriage as by definition that has to occur early in pregnancy (i.e. prior to 20-24 weeks of gestation in the different populations included in the analyses).”

***In S7 Table,*** we removed information on stillbirth and miscarriage history.

In particular, I think it's necessary for the authors to fully address the concerns raised by Reviewer 2 regarding possible reverse causation, pleiotropy and shared aetiology. Some of the causal claims should also be toned down a bit.

Thank you for the suggestions. We have amended the manuscript as suggested by Reviewer 2 (pages 13-16). We have toned down our statements following the requests from the editors (pages 4, 5, 7 and 8).

Requests from the editors:

General comments:

Please include line numbers in your revised manuscript, ideally not starting from 1 with each new page.

Your study is observational and therefore causality cannot be inferred. Please avoid use of causal language such as ‘effect’, referring to ‘associations’ instead. Please replace ‘causal effects’ with ‘causal associations’ throughout your manuscript.

Throughout the paper, please adapt reference call-outs to the following style: "... risks of gestational diabetes (GD) [5,6]..." (noting the absence of spaces within the square brackets).

Thank you for the suggestions. We have added the line number, replaced ‘effects’ with ‘associations’ (pages 4, 5, 7 and 8), and amended the reference style. Please see the revised manuscript.

Title: Please revise your title according to PLOS Medicine's style. Please place the study design in the subtitle (ie, after a colon). We suggest “Association between insomnia and pregnancy and perinatal outcomes: A Mendelian randomization analysis” or similar

We have revised our title.

(page 1, line 1) From: “Associations of insomnia with pregnancy and perinatal outcomes: Findings from Mendelian randomization and conventional observational studies in up to 356,069 women”

To: “Associations between insomnia and pregnancy and perinatal outcomes: Mendelian randomization and conventional observational analyses”

Abstract Background:

Please avoid use of causal language. Please revise to “Our aim was to estimate the association between insomnia and stillbirth, miscarriage, …”

We have revised our aim as suggested.

(page 2, line 22) From: “Our aim was to test whether insomnia causes stillbirth … or low/high offspring birthweight”

To: “Our aim was to estimate the association of insomnia with stillbirth … and low/high offspring birthweight”

Abstract Methods and Findings:

Please revise to “IVW showed evidence of an association between…”

Please revise to “For other outcomes IVW indicated potentially clinically important associations…”

Please provide brief demographic details of the study population (e.g. age, ethnicity, etc)

We have made the following changes:

(page 2, line 32) From: “IVW showed evidence of an effect of genetic susceptibility to insomnia on miscarriage”

To: “IVW showed evidence of an association of genetic susceptibility to insomnia with miscarriage”

(page 2, line 35) From: “For other outcomes IVW indicated potentially clinically important adverse effects of insomnia …”

To: “For other outcomes IVW indicated potentially unfavourable associations of insomnia …”

(page 2, line 26) From: “We used data (N=356,069) from the UK Biobank, FinnGen, and three European birth cohorts (Avon Longitudinal Study of Parents and Children (ALSPAC), Born in Bradford, and Norwegian Mother, Father and Child Cohort Study).”

To: “We used data from women of European descent (N=356,069, mean ages at delivery 25.5-30.0 years) from UK Biobank, FinnGen, Avon Longitudinal Study of Parents and Children (ALSPAC), Born in Bradford, and the Norwegian Mother, Father and Child Cohort.”

Abstract Conclusions:

Please begin your Abstract Conclusions with "In this study, we observed ..." or similar, to summarize the main findings from your study, without overstating your conclusions. Please emphasize what is new and address the implications of your study more specifically, being careful to avoid assertions of primacy. Please revise to “In this study, we observed evidence of causal associations…”

We have made the following changes:

(page 3, line 45) From: “There is evidence of causal effects of insomnia on miscarriage, perinatal depression, and LBW.”

To: “In this study, we observed evidence of causal associations of insomnia with miscarriage, perinatal depression, and LBW.”

(page 3, line 46) From: “We highlight the need for larger studies with genomic data and pregnancy outcomes.”

To: “Our findings highlight the importance of improving healthy sleep in women of reproductive age, though larger studies are needed for replication.”

Author Summary:

Please temper assertions of primacy by adding ‘to the best of our knowledge’ or similar to the third bullet point of ‘Why was this study done?’

In the final bullet point of ‘What Do These Findings Mean?’, please describe the main limitations of the study in non-technical language.

We have made the following changes:

(page 4, line 56) From: “No Mendelian randomization has been conducted to explore the association of insomnia with adverse pregnancy and perinatal outcomes.”

To: “To the best of our knowledge, no Mendelian randomization (MR) has been conducted to explore the association of insomnia with adverse pregnancy and perinatal outcomes.”

(page 5, line 75) We added: “Key limitations of our study are potential horizontal pleiotropy (particularly for perinatal depression) and low statistical power in MR, and residual confounding in multivariable regression. Replication in larger MR studies would be valuable.”

Introduction:

Please temper assertions of primacy by adding ‘to the best of our knowledge’ or similar to the penultimate paragraph. Please replace ‘causal effects’ with ‘causal associations’

We have made the following changes:

(page 7, line 119) From: “MR can be used to estimate a causal effect from the associations of the SNPs with the exposure and outcome.”

To: “MR can be used to estimate a causal association from SNP-exposure and SNP-outcome associations, if the underlying assumptions (in Discussion) are true.”

(page 7, line 123) From: “This approach has previously been used to evaluate causal effects of self-reported insomnia on risks of type 2 diabetes … but not pregnancy and perinatal outcomes.”

To: “This approach has previously been used to evaluate causal associations of insomnia with type 2 diabetes … but to the best of our knowledge not pregnancy/perinatal outcomes.”

(page 7, line 127) From: “The aims of this study are to (I) explore the causal effects of maternal genetic susceptibility to insomnia on stillbirth …”

To: “The aims of this study are to (I) explore the causal associations of maternal genetic susceptibility to insomnia with stillbirth …”

Methods:

Did your study have a prospective protocol or analysis plan? Please state this (either way) early in the Methods section. If a prospective analysis plan (from your funding proposal, IRB or other ethics committee submission, study protocol, or other planning document written before analyzing the data) was used in designing the study, please include the relevant prospectively written document with your revised manuscript as a Supporting Information file to be published alongside your study, and cite it in the Methods section. A legend for this file should be included at the end of your manuscript. If no such document exists, please make sure that the Methods section transparently describes when analyses were planned, and if/when reported analyses differed from those that were planned. Changes in the analysis-- including those made in response to peer review comments-- should be identified as such in the Methods section of the paper, with rationale. If a reported analysis was performed based on an interesting but unanticipated pattern in the data, please be clear that the analysis was data-driven.

We do not have a prospective analysis plan specific to this study. We have declared the changes in response to academic editor (page 1) and reviewer 2 (pages 14-15).

***In Study population,***

(page 9, line 145) We added: “We did not have a pre-specified analysis plan.”

Please ensure that the study is reported according to the STROBE-MR guideline, and include the completed STROBE-MR checklist as Supporting Information. Please add the following statement, or similar, to the Methods: "This study is reported as per the Strengthening the Reporting of Observational Studies in Epidemiology (STROBE) guideline, specific for mendelian randomization (S1 Checklist)." The STROBE-MR guideline can be found at <https://www.strobe-mr.org/>

We have updated the STROBE checklist we used to the newly published STROBE-MR checklist [6]. We have also made the following change in methods:

We added: “This study is reported as per the Strengthening the Reporting of Observational Studies in Epidemiology (STROBE) guideline, specific for MR (S1 Checklist) [6].”

Results:

For the ORs presented, please specify the comparison group.

Please be clear whether the results presented in the main text are adjusted analyses and, if so, please indicate which factors are adjusted for.

We have made the following changes:

(page 17, line 299) From: “… and 32 (OR 1.02, 95% CI: 1.01, 1.03) weeks of gestation in ALSPAC … GWAS of UKB plus 23andMe women (OR 1.05, 95% CI: 1.05, 1.06).”

To: “… and 32 (OR 1.02 per effect allele, 95% CI: 1.01, 1.03) weeks of gestation in ALSPAC … GWAS of UKB plus 23andMe women (OR 1.05 per effect allele, 95% CI: 1.05, 1.06).”

(page 17, line 305) From: “In the analysis combining all cohorts, lifetime tendency to insomnia was associated with …”

To: “In MR IVW combining all cohorts, lifetime susceptibility to insomnia (vs no insomnia) was associated with …”

(page 17, line 309) From: “The three that did not include the null were miscarriage (OR 1.60, 95% CI: 1.18, 2.17), perinatal depression (OR 3.56, 95% CI: 1.49, 8.54) and LBW (OR 3.17, 95% CI: 1.69, 5.96).”

To: “The three that did not include the null using IVW were miscarriage, perinatal depression and LBW (Fig 2).”

(page 20, line 342) From: “After adjusting for potential confounders, there were associations of insomnia at 18 and 32 weeks of gestation with stillbirth history, miscarriage history (only assessed for 18 weeks), GD (32 weeks only), HDP and perinatal depression, with similar magnitudes of association to those seen for the MR analyses, and with imprecision meaning some 95% CIs included the null (Fig 3).”

To: “After adjusting for maternal age, education, BMI, smoking, alcohol intake and household occupational social class, there were associations of insomnia at 18 weeks of gestation with HDP and perinatal depression (Fig 3). They were in similar magnitudes to those seen in MR, but with imprecision meaning some 95% CIs included the null.”

(page 20, line 347) From: “Associations with stillbirth history (OR for insomnia at 18 weeks 2.10, 95% CI: 1.20, 3.69), miscarriage history (OR for insomnia at 18 weeks 1.30, 95% CI: 1.12, 1.51), and perinatal depression (OR for insomnia at 18 weeks 2.96, 95% CI: 2.42, 3.63), were the most reliable with CIs that did not include the null (Fig 3).”

To: “Associations with perinatal depression (e.g. OR for insomnia (vs no insomnia) 2.96, 95% CI: 2.42, 3.63) were the most strongest with CIs not including the null (Fig 3).”

Discussion:

Please present and organize the Discussion as follows: a short, clear summary of the article's findings; what the study adds to existing research and where and why the results may differ from previous research; strengths and limitations of the study; implications and next steps for research, clinical practice, and/or public policy; one-paragraph conclusion.

Please remove all subheadings within your Discussion e.g. Study strengths and limitations

Please avoid causal language such as “effect” and “increased risk”. For example, please revise to “the strongest association using MR evidence was observed between insomnia and the risk of miscarriage, …” or similar.

Please temper assertions of primacy by adding ‘to the best of our knowledge’ or similar to “our novel finding” and “it is the first study to use MR…”

Thank you for the suggestions. We have made the following changes:

(page 23, line 361) From: “To our knowledge this is the first MR study …”

To: “To the best of our knowledge, this is the first MR study …”

(page 23, line 370) From: “our MR results provide evidence that a lifetime tendency to insomnia may increase the risk of stillbirth …”

To: “our MR results provide evidence that a lifetime susceptibility to insomnia might associate with higher risks of stillbirth …”

(page 23, line 373) We deleted: “However, we acknowledge that MR analyses are statistically inefficient … all but three outcomes having 95% CI that included the null.”

(page 23, line 375) From: “Thus, we have strongest MR evidence for insomnia increasing the risk of miscarriage, perinatal depression, and LBW.”

To: “Given all but three of these outcomes had 95% CIs that included the null, we have relatively strong statistical evidence from MR for lifelong susceptibility to insomnia associating with miscarriage, perinatal depression, and LBW.”

(page 23, line 382) From: “Our findings in both MR and multivariable regression of increased risks of perinatal depression …”

To: “Our findings in both MR and multivariable regression of an association of insomnia with perinatal depression …”

(page 24, line 387) From: “… recent systematic reviews have only identified one cross-sectional study (N=222) of the association of insomnia with stillbirth [2,7].”

To: “… recent systematic reviews have only identified one cross-sectional study of the association of insomnia with stillbirth [2,7]. This cross-sectional study compared outcomes between 190 women reporting poor sleep quality and 30 women who did not, and found no association with stillbirth, although this was not the main focus of the paper [8].”

(page 24, line 392) From: “Thus, our novel findings of a possible effect of insomnia on miscarriage in both MR and multivariable regression analyses warrants replication.”

To: “Thus, our novel finding of a possible association of insomnia with miscarriage in MR warrants replication, and larger studies that support analyses with both miscarriage and stillbirth would be valuable.”

(page 24, line 405) From: “… including insomnia resulting in increased risks of adiposity, insulin resistance and other cardiometabolic outcomes that could then influence related pregnancy outcomes (GD, HDP, LBW and HBW) and influence placentation and hence miscarriage, stillbirth and PTB.”

To: “… including insomnia resulting in increased risks of adiposity and insulin resistance that could then influence related pregnancy outcomes (GD, HDP and HBW). Insomnia has also been suggested to influence maternal blood pressure and placental function which in turn would increase risks of HDP, miscarriage, stillbirth and PTB.”

(page 24, line 409) From: “MR analyses support effects of insomnia on coronary heart disease …”

To: “MR analyses support causal associations of insomnia with coronary heart disease …”

(page 25, line 413) From: “Similarly, MR analyses have found a potential effect of insomnia on depressive symptoms …”

To: “Similarly, MR analyses have found a causal association of insomnia on depressive symptoms …”

(page 25, line 416) We deleted: “Study strengths and limitations”

(page 25, line 417) From: “(I) it is the first study to use MR to explore potential effects of insomnia on pregnancy and perinatal outcomes”

To: “(I) to the best of our knowledge, it is the first study to use MR to explore associations of insomnia with pregnancy and perinatal outcomes”

(page 25, line 422) We deleted: “To our knowledge, this is the first study to explore associations with miscarriage and stillbirth, using either multivariable regression or MR.”

(page 26, line 436) From: “Interpretation of the effect estimates from our MR analyses …”

To: “Interpretation of our MR estimates …”

(page 28, line 491) From: “In conclusion, our study raises the possibility of adverse causal effects of insomnia on miscarriage, perinatal depression, and LBW.”

To: “In conclusion, our study raises the possibility of unfavourable associations of insomnia with miscarriage, perinatal depression and LBW.”

Figures:

Please consider avoiding the use of red and green in order to make your figure more accessible to those with colour blindness.

Thank you for pointing this out. We have removed colours, and used different points shapes for different associations to make sure they can be discriminated in grayscale figures.

Tables:

When a p value is given, please specify the statistical test used to determine it in the table legend.

We have made the following changes:

***In S3 Table,*** we added: “Fixed-effects meta-analysis of logistic regression estimates in UKB and 23andMe, using SNP P-values weighted by sample size.”

***In S6 Table,*** from: “^b^ P-value for Q <0.05 suggests between-SNP heterogeneity.”

To: “^b^ P-value for Cochran’s Q-statistic in IVW and MR-Egger testing statistical evidence for between-SNP heterogeneity.”

From: “^c^ P-value for intercept <0.05 suggests unbalanced horizontal pleiotropy.”

To: “^c^ P-value for intercept from MR-Egger testing statistical evidence for unbalanced horizontal pleiotropy.”

References:

Where website addresses are cited, please specify the date of access.

Thank you for pointing this out. We have added the date of access in the reference list.

Comments from the reviewers:

Reviewer #1: This study examined the effects of insomnia on pregnancy and perinatal outcomes using two-sample Mendelian randomization design. It is a well-designed and carefully conducted study. Here are my specific comments on this study.

1. Please provide line number for easier reference.

Thank you for the suggestion. We have added the line number in our revised manuscript.

2. There is competing interest that needs to be carefully reviewed

Two authors (KT and DAL) declare conflicts of interest, which are consistent with their most recent paper at *PLoS Med* [9]. These were in the original submission. We believe these are indirect as neither are related to the content of this paper and so are unsure what the reviewer feels needs further review here. We are happy to provide any more details that the editor might wish to see.

3. The data are not fully available to the public.

For MR, we have released all summary level data that we generated in S4 & S5 Tables; summary level data from FinnGen are publicly available. We are not allowed (under clear data protection and governance regulations) to provide individual level data from the cohort studies (UKB, ALSPAC, BiB and MoBa), but have provided information on how access to those data can be obtained in our original submission. We have added more details as below.

***In Data Availability Statement,***

(page 29, line 500) We added: “The data in UKB, ALSPAC, BiB and MoBa are fully available via managed systems to any researchers. The managed system for these studies is a requirement of the study funders but access is not restricted on the basis of overlap with other applications to use the data or on the basis of peer review of the proposed science. Researchers have to pay for a dataset to be prepared for them.”

4. page 5, Introduction: "three RCT assessing the effects… or perinatal outcomes". I feel that this part can be briefer. No need to show the numbers here. Or you can discuss the results in more details in the discussion section.

Thank you for pointing this out. We have simplified this paragraph as below:

***In Introduction,***

(page 7, line 107) We deleted: “The first RCT reported a difference in mean score of -0.21 (95% confidence interval (CI): -0.30, -0.11) in 208 randomized women, with equivalent results for the other two being -0.24 (95% CI: -0.67, 0.19, N=194) and 0.34 (95% CI: -0.26, 0.93, N=91) [10-12].”

***In Discussion,***

(page 23, line 384) From: “… and with RCTs suggesting that pregnancy interventions to reduce insomnia decrease perinatal depression”

To: “… and with RCTs suggesting that pregnancy intervention with cognitive behavioural therapy to reduce insomnia decreases perinatal depression”

5. Page 8, outcome measures: "if multiple pregnancies were enrolled in the birth cohorts, we randomly selected one pregnancy per woman". First, how many multiple pregnancies are there in the dataset? Second, is this the best way to deal with multiple pregnancies? Can you adjust for the multiple pregnancies using techniques such as random effect model? Also, if you have to choose one of the pregnancies, is that better to choose the first pregnancy rather than randomly select one pregnancy. I assume most the single pregnancies are the first pregnancy?

Thank you for the suggestion. Women having multiple pregnancies constituted 0.8%, 10.6% and 18.4% of women in ALSPAC, BiB and MoBa, respectively. Choosing the first pregnancy could introduce selection bias towards younger age, lower parity and higher morbidity of HDP [13]. Thus, we followed EGG consortium [14] to not select primiparous birth to avoid potential selection bias. For each outcome, women having multiple pregnancies had limited contribution to our overall sample combining UKB, FinnGen (where possible), ALSPAC, BiB, MoBa (all <5%), and cannot dominate our results. We have revised our manuscript as below:

(page 10, line 170) From: “If multiple pregnancies were enrolled in the birth cohorts, we randomly selected on pregnancy per woman.”

To: “There were a small proportion of women who contributed more than one pregnancy (<5% of total N for each outcome). To avoid potential selection bias [15], we randomly selected one pregnancy per woman.”

6. Page 9, insomnia measures: The definitions of insomnia in ALSPAC and in other studies are different. I would say lifelong exposure to insomnia and insomnia during pregnancy are drastically different. Is that possible to adjust for the two measurements to make them more consistent with each other? For example, using studies collecting data for both definitions to examine the relationship between them and adjust the data of two definitions accordingly. Given the difference between the two definitions, I would say that comparison between the MR and conventional approach become less useful.

Thank you for pointing this out. We have removed our multivariable regression results on pregnancy loss history, as suggested by the academic editor (page 1). We agree that MR estimates might be larger than those from multivariable regression [16]. However, we still feel, as in other papers exploring pregnancy health and triangulating MR with multivariable regression evidence [17-20], such comparison could be useful due to their different sources of bias [21,22]. We cannot model repeated measures of women’s insomnia across adulthood because of no data. We have clarified this in the discussion.

(page 25, line 419) From: “(II) we compared those MR findings with multivariable regression results of insomnia symptoms in pregnancy, adjusting for a priori defined key confounders;”

To: “(II) we conducted confounder-adjusted multivariable regression of insomnia in pregnancy in ALSPAC – a larger sample than most previous studies.”

(page 27, line 474) We added: “As the sources of bias in our two methods (MR and multivariable regression) differ, where we have consistency between them this increases our confidence in those consistent results being the causal association [17-21].”

(page 27, line 476) From: “The similarity of the multivariable regression and MR results for miscarriage, stillbirth, GD, HDP and perinatal depression suggests residual confounding is unlikely to have biased regression results for these outcomes.”

To: “The similarity of the multivariable regression and MR results for GD, HDP and perinatal depression suggests it is unlikely that residual confounding has biased regression results, horizontal pleiotropy has substantially affected MR results, or different sources of selection bias in the two have a strong impact, for these outcomes.”

(page 27, line 480) From: “The attenuated to the null associations for preterm birth, LBW and HBW compared to MR results suggests possible masked confounding or other biases specifically affecting these but not other outcomes.”

To: “The associations for PTB, LBW and HBW were attenuated to the null compared to MR results. These suggest possible masking confounder, other biases specific to the multivariable regression, or that the MR is estimating an accumulative effect of insomnia across the lifecourse [16], whereas the observational analyses reflect exposure only from 18 weeks of gestation to occurrence of outcome.”

7. Page 9, SNP selection and SNP-insomnia associations: fitting linear regression on a binary outcome (i.e., insomnia) is inappropriate. Can you fit logistic or probit model and predict the log-odds of insomnia instead? I don't see why you have to worry about non-collapsibility and interpretation here. This are intermediate estimates anyway.

Thank you for pointing this out. We calculated SNP-insomnia associations in ALSPAC using logistic regression to compare with the ORs reported by the GWAS [23]. If log(OR) was used as the denominators in MR, estimates were per 1 unit change in insomnia on log odds scale. This unit would be difficult to interpret [24], and less comparable to multivariable regression in ALSPAC (reporting ORs on risk difference scale – comparing having insomnia to not). As such, we followed Sander Greenland’s work [25] to generate SNP-insomnia associations in linear regression for MR. We have amended our manuscript to clarify this point.

***In Methods,***

(page 12, line 12) From: “We fitted linear regression to individual-level data from 208,140 UKB women to recalculate SNP-insomnia associations for two-sample MR analyses, to avoid non-collapsibility of ORs and difficult interpretation of MR estimates’ unit [24].”

To: “We followed the standard IV approach [25] first using linear regression with individual-level data from 208,140 UKB women to obtain SNP-insomnia association summary data for two-sample MR analyses. This provides estimates on the risk difference scale, which is more interpretable and comparable to our multivariable regression results [24].”

***In Fig 2 and S2 Fig,***

From: “Odds ratio (OR, 95% confidence interval (CI))”

To: “Odds ratio (OR, 95% confidence interval (CI)) for lifelong susceptibility to insomnia (yes vs no)”

***In Fig 3,***

From: “Odds ratio (OR, 95% confidence interval (CI))”

To: “Odds ratio (OR, 95% confidence interval (CI)) for self-report insomnia (yes vs no) during the index pregnancy”

8. Page 13: "if the MR-Egger intercept is not null it suggests the presence of horizontal pleiotropy, and the MR-Egger slope provides an effect estimate corrected for unbalanced horizontal pleiotropy" Then, why not use the MR-Egger directly to avoid making assumption on horizontal pleiotropy? Given the sample size you have, do you really have to worry about statistical power here?

Thank you for raising these points. We only identified one P-value <0.05 among all 27 tests shown in S6 Table, which could be due to chance. We did not use MR-Egger slope as main result for perinatal depression, because it is known to be less efficient than IVW [26] and provides wide CIs overlapped with IVW results. We have mentioned MR-Egger results in results and toned down the conclusion regarding perinatal depression.

We have observed wide CIs (suggesting insufficient statistical power) in MR because of small case numbers. In total, we included 5027 stillbirth, 55,899 miscarriage, 6696 gestational diabetes, 9731 HDP, 6492 perinatal depression, 14,178 LBW and 3492 HBW cases. We have clarified this throughout the manuscript.

***In Abstract,***

(page 2, line 35) From: “For other outcomes IVW indicated potentially clinically important adverse effects of insomnia (OR range 1.20 to 2.43), but CIs were wide and included the null.”

To: “For other outcomes IVW indicated potentially unfavourable associations of insomnia (OR range 1.20 to 2.43), but 95% CIs were wide and included the null.”

(page 2, line 42) From: “Key limitations are potential horizontal pleiotropy and low statistical power in MR …”

To: “Key limitations are potential horizontal pleiotropy (particularly for perinatal depression) and low statistical power in MR …”

(page 3, line 45) From: “There is evidence of causal effects of insomnia on miscarriage, perinatal depression, and LBW.”

To: “In this study, we observed evidence of causal associations of insomnia with miscarriage, perinatal depression, and LBW.”

***In Author summary,***

(page 4, line 68) From: “We found consistent evidence from Mendelian randomization and multivariable regression that insomnia was associated with higher risks miscarriage and perinatal depression, and Mendelian randomization also suggested an unfavourable effect on low offspring birthweight.”

To: “We found consistent evidence from MR and multivariable regression that insomnia was associated with a higher risk of perinatal depression, and MR also suggested unfavourable associations with miscarriage and low offspring birthweight.”

(page 5, line 75) We added: “Key limitations of our study are potential horizontal pleiotropy (particularly for perinatal depression) and low statistical power in MR, and residual confounding in multivariable regression. Replication in larger MR studies would be valuable.”

***In Discussion,***

(page 23, line 23) From: “Thus, we have strongest MR evidence for insomnia increasing the risk of miscarriage, perinatal depression, and LBW.”

To: “Given all but three of these outcomes had 95% CIs that included the null, we have relatively strong statistical evidence from MR for lifelong susceptibility to insomnia associating with miscarriage, perinatal depression, and LBW.”

(page 23, line 382) From: “Our findings in both MR and multivariable regression of increased risks of perinatal depression …”

To: “Our findings in both MR and multivariable regression of an association of insomnia with perinatal depression …”

(page 25, line 413) From: “Similarly, MR analyses have found a potential effect of insomnia on depressive symptoms”

To: “Similarly, MR analyses have found a causal association of insomnia with depressive symptoms”

(page 25, line 430) We add: “Our results for perinatal depression require further validation using multivariable MR in a larger sample with more cases.”

(page 27, line 476) From: “The similarity of the multivariable regression and MR results for miscarriage, stillbirth, GD, HDP and perinatal depression suggests residual confounding is unlikely to have biased regression results for these outcomes.”

To: “The similarity of the multivariable regression and MR results for GD, HDP and perinatal depression suggests it is unlikely that residual confounding has biased regression results, horizontal pleiotropy has substantially affected MR results, or different sources of selection bias in the two have a strong impact, for these outcomes.”

(page 27, line 485) From: “Despite having a large sample size and our multivariable analyses being larger than most previous studies, several of our MR and multivariable regression estimates are imprecise.”

To: “MR analyses are statistically inefficient and despite combining relevant studies in order to increase sample size, several of our MR and multivariable regression estimates are imprecise due to small numbers of cases.”

(page 28, line 491) From: “In conclusion, our study raises the possibility of adverse causal effects of insomnia on miscarriage, perinatal depression, and LBW.”

To: “In conclusion, our study raises the possibility of unfavourable associations of insomnia with miscarriage, perinatal depression and LBW.”

Reviewer #2: The paper applies Mendelian randomization and multivariable linear regression to study the effects of lifetime tendency for insomnia on pregnancy related outcomes. Overall, I think this is a well written and presented study, and an interesting use of Mendelian randomization methods. There is some evidence of an effect of insomnia on some pregnancy outcomes, although many of the methods used are reasonably low powered and sensitivity analyses do not always support the primary analyses. Nonetheless, the authors place the results in the context of existing literature and their results provide the basis for future study of the identified associations. I have the following comments.

- Although it is noted in the Discussion, I feel that potential reverse causation could be particularly relevant here, especially given that in many cases it is unknown if the exposure occurred before or after the outcome. A bidirectional analysis and/or the MR Steiger test of directionality could be used to analyse potential reverse causation.

Thank you for the suggestion. We have removed our multivariable regression results on pregnancy loss history, as suggested by the academic editor (see response on page 1). For MR, we have added Steiger filtering [27]. We have amended our manuscripts.

***In Abstract,***

(page 2, line 35) From: “For other outcomes IVW indicated potentially clinically important adverse effects of insomnia …”

To: “For other outcomes IVW indicated potentially unfavourable associations of insomnia …”

***In Methods,***

(page 16, line 279) We added: “As suggested during peer review, we used Steiger filtering to identify SNPs explaining substantially more of the variance in an outcome than in insomnia (i.e. P-value <0.05) [27], and re-conducted MR IVW after removing those SNPs (listed in S3 Table).”

***In Results,***

(page 17, line 314) We added: “We further removed 26, 1 and 7 SNPs from analyses for stillbirth, perinatal depression and LBW, respectively (S3 Table), because Steiger filtering suggested these SNPs potentially more associated with the respective outcome than with susceptibility to insomnia (see Methods). MR IVW estimates were consistent for perinatal depression, slightly attenuated for LBW, and in the opposite direction for stillbirth with overlapped CIs both including the null (Fig 2).”

***In Discussion,***

(page 23, line 370) From: “our MR results provide evidence that a lifetime tendency to insomnia may increase the risk of stillbirth …”

To: “our MR results provide evidence that a lifetime susceptibility to insomnia might associate with higher risks of stillbirth …”

(page 27, line 469) We add: “Although we examined the possibility of reverse causality for individual SNPs using Steiger filtering in MR, this test could be influenced by measurement errors in insomnia and our outcomes, and by confounding with opposite directions for insomnia and the outcomes [27]. Multivariable regression results for maternal outcomes could also be vulnerable to reverse causality, as tendency towards the outcome might have influenced insomnia reported at 18 weeks of gestation.”

***In Fig 2,***

(page 19, line 330) We added: “^c^ After Steiger filtering, estimates were 0.83 (0.27, 2.54), 3.57 (1.49, 8.54) and 2.18 (1.13, 4.22) for stillbirth, perinatal depression and LBW, respectively.”

***In S3 Table,*** we added estimates from Steiger filtering.

We added: “^a^ To maximise statistical power, the variance of insomnia explained by each SNP (i.e. rsq.insomnia) was calculated based on SNP-insomnia associations from the UKB & 23andMe GWAS; the variance of each outcome explained by each SNP (i.e. rsq.outcome) was calculated based on SNP-outcome associations combining UKB, ALSPAC, BiB, MoBa and FinnGen (if available).”

We added: “^d^ This SNP was identified by Steiger filtering and removed for stillbirth in MR IVW as sensitivity analysis.”

We added: “^e^ This SNP was identified by Steiger filtering and removed for perinatal depression in MR IVW as sensitivity analysis.”

We added: “^f^ This SNP was identified by Steiger filtering and removed for LBW in MR IVW as sensitivity analysis.”

We added: “ALSPAC, Avon Longitudinal Study of Parents and Children; BiB, Born in Bradford; GD, gestational diabetes; GWAS, genome-wide association study; HBW, high offspring birthweight; HDP, hypertensive disorders of pregnancy; IVW, inverse variance weighted; LBW, low offspring birthweight; MoBa, Norwegian Mother, Father and Child Cohort Study; MR, Mendelian randomization; PTB, preterm birth.”

***In S5 Table,*** we added estimates from all UKB women, which were used in Steiger filtering.

We added: “^a^ SNP-outcome associations were meta-analysed using fixed-effects (with inverse variance weights), and then used in Steiger filtering.”

- The Discussion suggests there are known risk factors which may sit on pleiotropic pathways from the genetic instruments to the outcomes, and hence be causing bias in the MR results. Do the genetic instruments associate with any of these possibly pleiotropic risk factors? The conclusion regarding the effect of insomnia on perinatal depression seems somewhat weak: there is evidence of unbalanced pleiotropy from the MR-Egger intercept test, and the direction of the MR-Egger point estimate is inconsistent with the MR-IVW result. Could these results be explained by horizontal pleiotropy, and if so, could this be explored in a multivariable MR analysis?

Thank you for the suggestion. We agree that one of the MR-Egger intercept P-values <0.05 would weak our results for perinatal depression. We have mentioned MR-Egger results in results and toned down the conclusion regarding perinatal depression (response to reviewer 1 Q8 on pages 12-13). We identified associations of our insomnia SNPs with possible risk factors in our previous MR study, but we also found insufficient power to conduct MVMR [28]. We have added this point in the discussion.

(page 25, line 430) We added: “Our MR results for perinatal depression require further validation using multivariable MR in a larger sample with more cases.”

- Three SNPs were excluded from the BiB dataset due to having a minor allele frequency < 0.01. Does this indicate that the genetic distribution of individuals in BiB is not similar to the other cohorts? For example, rs10947428 has a minor allele frequency of approx 0.2 in the other datasets, which seems to be a big difference to BiB. Is it therefore valid to combine BiB with the other datasets? Also, rs9943753 is given in the text as a SNP which is excluded from BiB, but doesn't seem to be in the list of instruments in the supplementary tables.

BiB have contributed to published GWAS and MR studies [18,29,30], and thus is reliable and be comparable to other European cohorts. We removed two rare SNPs (rs10947428 and rs117037340) from MR analysis, as recommended by GWAS guideline [31]. For those SNPs included in MR analysis, BiB showed similar effect allele frequencies compared to UKB, ALSPAC and MoBa (S5 Table). The SNP rs9943753 was identified by the GWAS of insomnia [23], but removed due to linkage disequilibrium before MR. Therefore, rs9943753 was not listed in S3 Table. We have amended our manuscript.

(page 12, line 225) From: “Three SNPs (i.e. rs10947428, rs9943753, and rs117037340) were excluded from BiB analyses due to their minor allele frequency lower than 1%.”

To: “Two SNPs (rs10947428 and rs117037340) were excluded from BiB analyses due to their minor allele frequency lower than 1%.”

- For the leave-one-out analysis of cohorts shown in S2 Fig, it might be relevant to also do this analysis without UKB. UKB dominates the others (due to its size), so I don't think the analysis is able to show whether there is heterogeneity between the birth cohorts. This may, for example, help to show whether BiB is from a similar population group to the other cohorts or not.

Thank you for the suggestion. We have made the following changes.

***In Methods,***

(page 13, line 243) From: “… and used leave-one (study)-out analysis to assess the degree of heterogeneity between cohorts.”

To: “… and used leave-one (study)-out analysis (initially across all cohorts, and then among non-UKB cohorts during peer review) to assess the degree of heterogeneity between cohorts.”

***In Results,***

(page 17, line 311) From: “Results were broadly consistent, with the point estimates showing some difference with stillbirth, GD and PTB, though CIs were very wide for some outcomes.”

To: “Results were broadly consistent but dominated by large cohorts (e.g. UKB and FinnGen), with the point estimates inflated and very wide CIs in small birth cohorts.”

***In S2 Fig,*** we added a figure for leave-one (study)-out analysis without UKB.

From: “Leave-one (study)-out analyses for causal effects of insomnia on adverse pregnancy and perinatal outcomes in Mendelian randomization using inverse variance weighted estimates.”

To: “Leave-one (study)-out analyses of MR IVW estimates for causal associations of insomnia with adverse pregnancy and perinatal outcomes across (a) all cohorts and (b) non-UKB cohorts.”

We added: “IVW, inverse variance weighted; MR, Mendelian randomization”

- Page 13: "if the MR-Egger intercept is not null it suggests the presence of horizontal pleiotropy" - should "horizontal" be "unbalanced" in this sentence?

Yes, we have revised this sentence.

(page 15, line 266) From: “if the MR-Egger intercepts is not null it suggests the presence of horizontal pleiotropy”

To: “if the MR-Egger intercepts is not null it suggests the presence of unbalanced horizontal pleiotropy”

Reviewer #3: Thank you for the opportunity to review this manuscript. The paper addresses an important question, whether sleep disturbances during pregnancy is causally linked to adverse pregnancy outcomes. Insomnia is becoming increasingly common, and is recognized to be associated with a variety of adverse health outcomes. As highlighted by the authors, several epidemiological studies have highlighted potential links between sleep disorders including insomnia and pregnancy outcome, though whether insomnia is causally linked to these outcomes are not clear.

The authors sought to address this series of research question using several datasets, including UK Biobank, 3 birth cohorts (ALSPAC, BIB, MoBa) and FinnGen. Results obtained from the different datasets were then meta-analyzed, as appropriate. They also performed multivariable regression on association between insomnia, as reported at 18 weeks gestation and 32 weeks gestation, with different pregnancy outcome, with adjustment of the known confounders. The main findings were significant, and clinically relevant association between insomnia and miscarriage, perinatal depression, and low birthweight. Some of the sensitivity analyses gave additional support, though some of the other analyses had CI that overlapped with null.

The study is overall well-performed, and utilized several of the largest datasets available to address the research question. The outcome definitions have been harmonized. The MR methodology and sensitivity analyses are clearly described, as would be expected given the extensive expertise on MR from this group of investigators from Bristol.

Results from the main analysis (using IVW), and the sensitivity analyses using weighted median and MR-Egger, were mostly consistent in direction and size of effects. Perhaps rather surprisingly, the confidence interval for the insomnia-adverse pregnancy outcome and perinatal outcome analysis was quite wide for the majority of outcomes, despite the large sample size. The between-study heterogeneity appeared limited for most outcomes, suggesting the source of the variation is prob not due to study differences.

Some limitations include the timing of the assessment of insomnia, which was at 18 and 32weeks gestation in ALSPAC, but with history of stillbirth or history of miscarriage used as the outcome. Another major limitation is that insomnia was defined according to self-reported measures.

Major comments

1. The confidence intervals for the estimates were rather large for most outcomes examined despite the large sample size. Can the authors discuss and speculate more on why this would be the case?

Thank you for the suggestion. We observed wide CIs for most outcomes in MR because of their small numbers of cases: 5027 stillbirth, 6696 gestational diabetes, 9731 HDP, 6492 perinatal depression, 14,178 LBW and 3492 HBW. In multivariable regression, ALSPAC had very limited numbers of cases for gestational diabetes. We have presented the numbers of cases and controls in Table 1 (for MR) and Fig 3 (for multivariable regression). We have also amended our discussion as follows:

(page 25, line 430) We added: “Our MR results for perinatal depression required further validation using multivariable MR in a larger sample with more cases.”

(page 27, line 485) From: “Despite having a large sample size and our multivariable analyses being larger than most previous studies, several of our MR and multivariable regression estimates are imprecise.”

To: “MR analyses are statistically inefficient and despite combining relevant studies in order to increase sample size, several of our MR and multivariable regression estimates are imprecise due to small numbers of cases.”

2. In general, genetically determined exposures (e.g. insomnia in this case) would show larger effects by the instrumental variable (reflecting lifelong predisposition to the exposure) compared to effect size observed in the multivariable regression analysis. This was however not the case for the outcomes examined in this analysis. Is this related to the impact of the exposure being limited to that during pregnancy. Perhaps some brief discussion of this would be useful.

Thank you for pointing this out. We compared our MR (Fig 2) with multivariable regression in ALSPAC (Fig 3), and MR showed larger point estimates than multivariable regression estimates, despite wide CIs (Table below).

| Outcome | Our study | OR (95% CI) |
| --- | --- | --- |
| GD | MR IVW | 1.20 (0.52, 2.77) |
|  | Multivariable regression (18 weeks, adjusted) | 1.01 (0.42, 2.45) |
| HDP | MR IVW | 1.71 (0.75, 3.89) |
|  | Multivariable regression (18 weeks, adjusted) | 1.05 (0.89, 1.24) |
| Perinatal depression | MR IVW | 3.56 (1.49, 8.54) |
|  | Multivariable regression (18 weeks, adjusted) | 2.96 (2.42, 3.63) |
| PTB | MR IVW | 1.31 (0.54, 3.21) |
|  | Multivariable regression (18 weeks, adjusted) | 0.89 (0.65, 1.23) |
| LBW | MR IVW | 3.17 (1.69, 5.96) |
|  | Multivariable regression (18 weeks, adjusted) | 0.92 (0.69, 1.24) |
| HBW | MR IVW | 2.43 (0.81, 7.34) |
|  | Multivariable regression (18 weeks, adjusted) | 0.75 (0.46, 1.23) |

However, our MR and multivariable regression estimates for GD, HDP and PTB are smaller than those reported by previous systematic reviews [7,32]. We have amended our discussion as below:

(page 24, line 394) From: “Previous systematic reviews of observational associations of insomnia with GD, HDP and PTB have some consistency with our main MR results, but few observational studies investigated LBW or HBW [2,7].

To: “Previous systematic reviews of observational associations of insomnia with GD (OR 1.37, 95% CI: 1.12, 1.69), HDP (OR 1.72, 95% CI: 1.16, 2.56) and PTB (OR 1.49, 95% CI: 1.17, 1.90) are directionally consistent but with stronger ORs than our main MR results [7].”

(page 24, line 397) We added: “These stronger associations could be due to insufficient adjustment of potential confounders and reverse causality, as many cross-sectional studies and unadjusted associations were included in the meta-analyses.”

(page 24, line 400) We deleted: “For example, ORs from meta-analyses for GD (1.37 combining 8 studies vs 1.20 in MR), HDP (1.72 combining 2 studies vs 1.71 in MR), PTB (1.49 combining 8 studies vs 1.31 in MR) were very similar to our MR analyses [7], despite the differing definitions of insomnia and outcomes and different assumptions for MR and multivariable regression [21].”

(page 27, line 476) From: “The similarity of the multivariable regression and MR results for miscarriage, stillbirth, GD, HDP and perinatal depression suggests residual confounding is unlikely to have biased regression results for these outcomes.”

To: “The similarity of the multivariable regression and MR results for GD, HDP and perinatal depression suggests it is unlikely that residual confounding has biased regression results, horizontal pleiotropy has substantially affected MR results, or different sources of selection bias in the two have a strong impact, for these outcomes.”

(page 27, line 480) From: “The attenuated to the null associations for preterm birth, LBW and HBW compared to MR results suggests possible masked confounding or other biases specifically affecting these but not other outcomes.”

To: “The associations for PTB, LBW and HBW were attenuated to the null compared to MR results. These suggest possible masking confounder, other biases specific to the multivariable regression, or that the MR is estimating an accumulative effect of insomnia across the lifecourse [16], whereas the observational analyses reflect exposure only from 18 weeks of gestation to occurrence of outcome.”

References:

1.	Sedov ID, Cameron EE, Madigan S, Tomfohr-Madsen LM. Sleep quality during pregnancy: A meta-analysis. Sleep Med Rev. 2018; 38168-76.

2.	Warland J, Dorrian J, Morrison JL, O'Brien LM. Maternal sleep during pregnancy and poor fetal outcomes: A scoping review of the literature with meta-analysis. Sleep Med Rev. 2018; 41197-219.

3.	Cronin RS, Li M, Thompson JMD, Gordon A, Raynes-Greenow CH, Heazell AEP, et al. An Individual Participant Data Meta-analysis of Maternal Going-to-Sleep Position, Interactions with Fetal Vulnerability, and the Risk of Late Stillbirth. EClinicalMedicine. 2019; 1049-57.

4.	Anderson NH, Gordon A, Li M, Cronin RS, Thompson JMD, Raynes-Greenow CH, et al. Association of Supine Going-to-Sleep Position in Late Pregnancy With Reduced Birth Weight: A Secondary Analysis of an Individual Participant Data Meta-analysis. JAMA Netw Open. 2019; 2(10):e1912614.

5.	Pengo MF, Won CH, Bourjeily G. Sleep in Women Across the Life Span. Chest. 2018; 154(1):196-206.

6.	Skrivankova VW, Richmond RC, Woolf BAR, Davies NM, Swanson SA, VanderWeele TJ, et al. Strengthening the reporting of observational studies in epidemiology using mendelian randomisation (STROBE-MR): explanation and elaboration. Bmj. 2021; 375n2233.

7.	Lu Q, Zhang X, Wang Y, Li J, Xu Y, Song X, et al. Sleep disturbances during pregnancy and adverse maternal and fetal outcomes: a systematic review and meta-analysis. Sleep Med Rev. 2020; 58101436.

8.	Owusu JT, Anderson FJ, Coleman J, Oppong S, Seffah JD, Aikins A, et al. Association of maternal sleep practices with pre-eclampsia, low birth weight, and stillbirth among Ghanaian women. Int J Gynaecol Obstet. 2013; 121(3):261-5.

9.	Millard LAC, Tilling K, Gaunt TR, Carslake D, Lawlor DA. Association of physical activity intensity and bout length with mortality: An observational study of 79,503 UK Biobank participants. PLoS Med. 2021; 18(9):e1003757.

10.	Felder JN, Epel ES, Neuhaus J, Krystal AD, Prather AA. Efficacy of Digital Cognitive Behavioral Therapy for the Treatment of Insomnia Symptoms Among Pregnant Women: A Randomized Clinical Trial. JAMA Psychiatry. 2020; 77(5):484-92.

11.	Manber R, Bei B, Simpson N, Asarnow L, Rangel E, Sit A, et al. Cognitive Behavioral Therapy for Prenatal Insomnia: A Randomized Controlled Trial. Obstet Gynecol. 2019; 133(5):911-9.

12.	Kalmbach DA, Cheng P, O'Brien LM, Swanson LM, Sangha R, Sen S, et al. A randomized controlled trial of digital cognitive behavioral therapy for insomnia in pregnant women. Sleep Med. 2020; 7282-92.

13.	Hernández-Díaz S, Toh S, Cnattingius S. Risk of pre-eclampsia in first and subsequent pregnancies: prospective cohort study. Bmj. 2009; 338b2255.

14.	Beaumont RN, Warrington NM, Cavadino A, Tyrrell J, Nodzenski M, Horikoshi M, et al. Genome-wide association study of offspring birth weight in 86 577 women identifies five novel loci and highlights maternal genetic effects that are independent of fetal genetics. Hum Mol Genet. 2018; 27(4):742-56.

15.	Hughes RA, Davies NM, Davey Smith G, Tilling K. Selection Bias When Estimating Average Treatment Effects Using One-sample Instrumental Variable Analysis. Epidemiology. 2019; 30(3):350-7.

16.	Sanderson E, Glymour MM, Holmes MV, Kang H, Morrison J, Munafò MR, et al. Mendelian randomization. Nature Reviews Methods Primers. 2022; 2(1):6.

17.	Richmond RC, Timpson NJ, Felix JF, Palmer T, Gaillard R, McMahon G, et al. Using Genetic Variation to Explore the Causal Effect of Maternal Pregnancy Adiposity on Future Offspring Adiposity: A Mendelian Randomisation Study. PLoS Med. 2017; 14(1):e1002221.

18.	Bond TA, Richmond RC, Karhunen V, Cuellar-Partida G, Borges MC, Zuber V, et al. Exploring the causal effect of maternal pregnancy adiposity on offspring adiposity: Mendelian randomisation using polygenic risk scores. BMC Med. 2022; 20(1):34.

19.	Hernáez Á, Rogne T, Skåra KH, Håberg SE, Page CM, Fraser A, et al. Body mass index and subfertility: multivariable regression and Mendelian randomization analyses in the Norwegian Mother, Father and Child Cohort Study. Hum Reprod. 2021; 36(12):3141-51.

20.	Magnus MC, Miliku K, Bauer A, Engel SM, Felix JF, Jaddoe VWV, et al. Vitamin D and risk of pregnancy related hypertensive disorders: mendelian randomisation study. BMJ. 2018; 361k2167.

21.	Lawlor DA, Tilling K, Davey Smith G. Triangulation in aetiological epidemiology. Int J Epidemiol. 2016; 45(6):1866-86.

22.	Munafò MR, Davey Smith G. Robust research needs many lines of evidence. Nature. 2018; 553(7689):399-401.

23.	Jansen PR, Watanabe K, Stringer S, Skene N, Bryois J, Hammerschlag AR, et al. Genome-wide analysis of insomnia in 1,331,010 individuals identifies new risk loci and functional pathways. Nat Genet. 2019; 51(3):394-403.

24.	Burgess S, Labrecque JA. Mendelian randomization with a binary exposure variable: interpretation and presentation of causal estimates. Eur J Epidemiol. 2018; 33(10):947-52.

25.	Greenland S. An introduction to instrumental variables for epidemiologists. Int J Epidemiol. 2000; 29(4):722-9.

26.	Burgess S, Davey Smith G, Davies NM, Dudbridge F, Gill D, Glymour MM, et al. Guidelines for performing Mendelian randomization investigations. Wellcome Open Res. 2019; 4186.

27.	Hemani G, Tilling K, Davey Smith G. Orienting the causal relationship between imprecisely measured traits using GWAS summary data. PLoS Genet. 2017; 13(11):e1007081.

28.	Yang Q, Sanderson E, Tilling K, Borges MC, Lawlor DA. Exploring and mitigating potential bias when genetic instrumental variables are associated with multiple non-exposure traits in Mendelian randomization. medRxiv. 2019 [cited 1 January 2021]. Available from: <https://www.medrxiv.org/content/10.1101/19009605v1>.

29.	Pervjakova N, Moen GH, Borges MC, Ferreira T, Cook JP, Allard C, et al. Multi-ancestry genome-wide association study of gestational diabetes mellitus highlights genetic links with type 2 diabetes. Hum Mol Genet. 2022.

30.	Brand JS, Gaillard R, West J, McEachan RRC, Wright J, Voerman E, et al. Associations of maternal quitting, reducing, and continuing smoking during pregnancy with longitudinal fetal growth: Findings from Mendelian randomization and parental negative control studies. PLoS Med. 2019; 16(11):e1002972.

31.	Marees AT, de Kluiver H, Stringer S, Vorspan F, Curis E, Marie-Claire C, et al. A tutorial on conducting genome-wide association studies: Quality control and statistical analysis. Int J Methods Psychiatr Res. 2018; 27(2):e1608.

32.	Zhang X, Zhang R, Cheng L, Wang Y, Ding X, Fu J, et al. The effect of sleep impairment on gestational diabetes mellitus: a systematic review and meta-analysis of cohort studies. Sleep Med. 2020; 74267-77.
