# Supplementary material for: Associations between insomnia and pregnancy and perinatal outcomes: Evidence from mendelian randomization and multivariable regression analyses
Source: PLoS Med. 2022 Sep 6;19(9):e1004090. doi: 10.1371/journal.pmed.1004090 (PMC9488815; doi:10.1371/journal.pmed.1004090)
Supplement: Attachment — Submitted filename: response letter.docx [file pmed.1004090.s017.docx]

Requests from the editors:

Points for a second major:

1. Response to reviewers: Please completely address the remaining points of reviewer 1.

Thank you for the reminder. Please see our responses to reviewer 1 on pages 9-11 of this document.

2. Title: We suggest: “Associations between insomnia and pregnancy and perinatal outcomes: Evidence from Mendelian randomization and observational analyses” or similar.

Thank you for the suggestion. We have amended the title.

(page 1, line 1) From: “Associations between insomnia and pregnancy and perinatal outcomes: Mendelian randomization and conventional observational analyses”

To: “Associations between insomnia and pregnancy and perinatal outcomes: Evidence from Mendelian randomization and multivariable regression analyses”

3. Abstract: Background: If feasible, it might be helpful to add a few words or a sentences to explain the gap in knowledge being addressed by your study, particularly the MR aspect (given that you point out previous work has already revealed observational evidence for associations between insomnia and adverse pregnancy/perinatal outcomes).

Thank you for the suggestion. We have added the research gap into the abstract.

(page 2, line 21) We add: “However, those associations could be vulnerable to residual confounding or reverse causality.”

4. Abstract: Methods and Findings: Please explicitly mention the main outcome measures.

We have listed the main outcomes in lines 22-24 (page 2).

5. Abstract: Methods and Findings: Please quantify the main results (with 95% CIs and p values).

We have made the following changes:

(page 2, line 33) From: “… miscarriage (odds ratio (OR): 1.60, 95% confidence interval (CI): 1.18, 2.17), perinatal depression (OR 3.56, 95% CI: 1.49, 8.54) and LBW (OR 3.17, 95% CI: 1.69, 5.96).”

To: “… miscarriage (odds ratio (OR): 1.60, 95% confidence interval (CI): 1.18, 2.17, p-value=0.002), perinatal depression (OR 3.56, 95% CI: 1.49, 8.54, p-value=0.004) and LBW (OR 3.17, 95% CI: 1.69, 5.96, p-value=0.0003).”

(page 2, line 43) From: “… perinatal depression (OR 2.96, 95% CI: 2.42, 3.63), but not with LBW (OR 0.92, 95% CI: 0.69, 1.24).

To: “… perinatal depression (OR 2.96, 95% CI: 2.42, 3.63, p-value=2×10^-25^), but not with LBW (OR 0.92, 95% CI: 0.69, 1.24, p-value=0.60).

6. Abstract: Line 33: Please define LBW at first use.

We have defined LBW in line 24 (page 2).

7. Abstract: Methods and Findings: Line 33-34: In light of the results, please revise the wording to: “For other outcomes IVW results did not support associations with insomnia, yielding observations that were directionally similar (OR range 1.20 to 2.43), but with 95% CIs that were wide and included the null.” or similar. It may also help to mention which specific outcomes are being described here, and please report OR, 95% CI, and p values for each.

We have rewritten this sentence.

(page 2, line 34) From: “For other outcomes IVW indicated potentially unfavourable associations of insomnia (OR range 1.20 to 2.43), but 95% CIs were wide and included the null.”

To: “IVW results did not support associations of insomnia with stillbirth, gestational diabetes, HDP, preterm birth and HBW, with wide CIs including the null.”

8. Abstract: Line 35: We suggest revising to: “Associations between genetically predicted insomnia and miscarriage, perinatal depression, and LBW were not observed in weighted median and MR Egger analyses. Results from these sensitivity analyses were directionally consistent with IVW analyses for all outcomes, with the exception of gestational diabetes, perinatal depression, and preterm birth.

Thank you for the suggestion. We have made the following changes.

(page 2, line 37) From: “Weighted median and MR Egger results were directionally consist, except for MR-Egger for gestational diabetes, perinatal depression, and preterm birth.”

To: “Associations of genetic susceptibility to insomnia with miscarriage, perinatal depression and LBW were not observed in weighted median or MR-Egger analyses. Results from these sensitivity analyses were directionally consistent with IVW results for all outcomes, with the exception of gestational diabetes, perinatal depression, and preterm birth in MR-Egger.”

9. Abstract: Methods and Findings: Would it be considered a limitation also that genetic instruments for insomnia were not specific to women in pregnancy (could there be different mechanisms related to pregnancy insomnia)?

Thank you for pointing this out. Our MR results reflect a lifetime susceptibility to insomnia, and thus should not be interpreted as estimates for maternal insomnia in pregnancy. Therefore, selection of genetic instruments from non-pregnant women would not be a key limitation of our MR study. We have included this point when mentioning further studies.

(page 3, line 51) From: “… though larger studies are needed for replication.”

To: “… though replication in larger studies, including with genetic instruments specific to insomnia in pregnancy.”

10. Abstract: Conclusions: Line 42: Please revise the wording to “...we observed some evidence supporting a possible causal relationship between genetically-predicted insomnia and miscarriage, perinatal depression, and LBW.” We suggest also mentioning that you also observed evidence supporting a relationship between insomnia at 18 weeks gestation and perinatal depression, but no observational evidence to support other outcomes.

We have made the following changes.

(page 3, line 47) From: “In this study, we observed evidence of causal associations of insomnia with miscarriage, perinatal depression, and LBW.”

To: “In this study, we observed some evidence supporting a possible causal relationship between genetically-predicted insomnia and miscarriage, perinatal depression, and LBW. Our study also supported a relationship between insomnia in pregnancy and perinatal depression, with no clear evidence of an association with LBW from multivariable regression.”

11. Abstract: Conclusions: Here and throughout (also Line 67-68), please rephrase, and avoid wording that implies “insomnia causes miscarriage” or similar.

Thank you for pointing these out. We have amended those sentences as suggested in the comments 10 and 14.

12. Author summary: Line 63: Please remove the word “consistent” given the findings from the various sensitivity analyses.

We have removed that word.

(page 4, line 73) From: “We found consistent evidence from MR and multivariable regression that …”

To: “We found evidence from MR and multivariable regression that …”

13. Author summary: Line 64-65: We suggest re-wording to: “...and MR analyses also suggested evidence for an association between genetically-predicted insomnia and risk of miscarriage and low offspring birthweight.”

We have amended the sentence as suggested.

(page 4, line 74) From: “… and MR also suggested unfavourable associations with miscarriage and low offspring birthweight.”

To: “… and MR analyses also suggested evidence for an association between genetically-predicted insomnia and risks of miscarriage and low offspring birthweight.”

14. Author summary: Line 67: We suggest: “These findings raise the possibility that interventions to improve healthy sleep may be beneficial for a healthy pregnancy.”

Thank you for the suggestion. We have amended the sentence.

(page 5, line 78) From: “Interventions to improve healthy sleep in women of reproductive age might be beneficial to a healthy pregnancy.”

To: “These findings raise the possibility that interventions to improve healthy sleep may be beneficial for a healthy pregnancy.”

15. Introduction: Line 89-90: Please clarify this sentence: “As disturbed sleep is a symptom of depression it is unclear whether these studies reflect a causal effect of insomnia, or it is part of the diagnostic criteria.”

We have written this sentence.

(page 6, line 100) From: “For example, all four studies included in the systematic review for perinatal depression were cross-sectional [1]. As disturbed sleep is a symptom of depression it is unclear whether these studies reflect a causal effect of insomnia, or it is part of the diagnostic criteria.”

To: “For example, all four studies included in the systematic review for perinatal depression were cross-sectional [1], in which disturbed sleep could be either a symptom of or a risk factor for depression.”

16. Methods: Line 130-133: Thank you for mentioning the lack of a pre-specified analysis plan. Please also make sure that the Methods section transparently describes when analyses were planned, and when/why any data-driven changes to analyses took place.

Thank you for pointing these out. We have added those details into the methods.

(page 8, line 141) From: “We did not have a pre-specified analysis plan.”

To: “This study was initiated using UKB in January 2018, with extra exploration of insomnia IVs and MR sensitivity analyses completed in February 2020 [2]. We searched for additional cohorts till July 2021, and harmonization across the cohorts had to be made continuously. Therefore, we did not have a pre-specified analysis plan.”

17. Results: Throughout this section, please present the OR, with 95% CIs and p values, for all main outcomes described.

We have made the following changes:

(page 16, 299) From: “The pooled 81 SNP-insomnia associations at 18 (OR 1.02 per effect allele, 95% CI: 1.01, 1.03) and 32 (OR 1.02 per effect allele, 95% CI: 1.01, 1.03) weeks of gestation in ALSPAC … UKB plus 23andMe women (OR 1.05 per effect allele, 95% CI: 1.05, 1.06).”

To: “The pooled 81 SNP-insomnia associations at 18 (OR 1.02 per effect allele, 95% CI: 1.01, 1.03, p-value=0.003) and 32 (OR 1.02 per effect allele, 95% CI: 1.01, 1.03, p-value=0.0004) weeks of gestation in ALSPAC … UKB plus 23andMe women (OR 1.05 per effect allele, 95% CI: 1.05, 1.06, p-value=4×10^-27^).”

(page 16, line 306) From: “… OR ranging from 1.20, for GD, to 3.56 for perinatal depression (Fig 2).”

To: “… OR ranging from 1.20 (95% CI: 0.52, 2.77, p-value=0.67) for GD, to 3.56 (95% CI: 1.49, 8.54, p-value=0.004) for perinatal depression (Fig 2).”

18. Results: Line 309: Please mention here that results of IVW analyses were attenuated for all associations identified in weighted median and MR-Egger analyses.

We have amended the results by adding this point.

(page 19, line 331) From: “Sensitivity analyses using weighted median and MR-Egger for all outcomes were directionally consistent, with the exception of MR-Egger results for GD, perinatal depression and PTB (Fig 2).”

To: “Sensitivity analyses using weighted median and MR-Egger were directionally consistent with IVW but attenuated to the null for all outcomes (except stillbirth), and MR-Egger results for GD, perinatal depression and PTB were attenuated to the null (Fig 2).”

19. Results: Lines 319-325: Please present the OR, 95% CIs, p values for associations with the primary outcomes in the text for the multivariable regression analyses.

We have made the following changes.

(page 19, line 347) From: “… there were associations of insomnia at 18 weeks of gestation with HDP and perinatal depression (Fig 3).”

To: “… there was an association of insomnia (vs no insomnia) at 18 weeks of gestation with perinatal depression (OR 2.96, 95% CI: 2.42, 3.63, p-value=2×10^-25^, Fig 3).”

(page 19, line 349) From: “They were in similar magnitudes to those seen in MR, but with imprecision meaning some 95% CIs included the null. Associations with perinatal depression (e.g. OR for insomnia (vs no insomnia) 2.96, 95% CI: 2.42, 3.63), were the most reliable with CIs not including the null (Fig 3).”

To: “Associations with other outcomes had imprecise 95% CIs including the null, although their point estimates were in similar magnitudes to those seen in MR (Fig 3).”

20. Results: Lines 314-317: Please describe in the text, or in the S6 Fig legend, how it was established whether adjustment for fetal genotype did or did not significantly alter SNP-outcome associations.

We have amended the S6 Fig legend by adding this point.

We add: “We consider a significant alteration of SNP-outcome associations if the slope is ≤0.8 or ≥1.2.”

21. Discussion: Line 340-348: Given the potential for this to be misinterpreted, we suggest rewording to clarify: “Overall, our MR results provide evidence that a lifetime susceptibility to insomnia might associate with higher risks of miscarriage, perintatal depression, and LBW. We did not observe evidence to support associations between genetically-predicted insomnia and stillbirth, GD, HDP, PTB, and HBW. In multivariable regression, we were unable to assess associations with miscarriage in the index pregnancy. Results for perinatal depression were consistent with the MR results, but this was not the case for LBW, for which no significant association with insomnia reported at 18 weeks gestation was observed.”

We have changed these sentences as suggested.

(page 22, line 373) From: “. Overall, our MR results provide evidence that a lifetime susceptibility to insomnia might associate with higher risks of stillbirth, miscarriage, GD, HDP, perinatal depression, PTB, LBW and HBW, with ORs of potential clinical importance (range 1.20 to 3.56) for all of these. Given all but three of these outcomes had 95% CIs that included the null, we have relatively strong statistical evidence from MR for lifelong susceptibility to insomnia associating with miscarriage, perinatal depression, and LBW.”

To: “Overall, our MR results provide evidence that a lifetime susceptibility to insomnia might associate with higher risks of miscarriage, perinatal depression, and LBW. We did not observe evidence to support associations between genetically-predicted insomnia and stillbirth, GD, HDP, PTB, and HBW.”

(page 22, line 379) From: “Result for perinatal depression replicated the MR results, but this was not the case for LBW, for which the ORs were much closer to the null.”

To: “Results for perinatal depression were consistent with the MR results, but this was not the case for LBW, for which no significant association with insomnia reported at 18 weeks gestation was observed.”

22. Discussion: Line 352-353: “Whilst previous studies have shown that pregnancy supine sleeping position (which is associated with insomnia) to be associated with stillbirth [6,39]” We suggest removing this, as per the discussion in the rebuttal letter.

We have amended this sentence as suggested.

(page 22, line 385) We delete: “Whilst previous studies have shown that pregnancy supine sleeping position (which is associated with insomnia) to be associated with stillbirth [3,4],”

23. Discussion: Line 387-388: “Results from these sensitivity analyses were broadly consistent with our main analyses but were less precise (i.e. had wider CIs).” Please provide a sentence or so of discussion on the implications of the fact that the results from the IVW analysis were not found to be robust in sensitivity analyses, in terms of how that affects your interpretations.

Thank you for the suggestion. We have made the following changes.

(page 24, line 420) From: “Results from these sensitivity analyses were broadly consistent with our main analyses but were less precise (i.e. had wider CIs). Our results for perinatal depression require further validation using multivariable MR in a larger sample with more cases.”

To: “Results from these sensitivity analyses were broadly consistent with the point estimates from IVW, but with wider 95% CIs given they were known to be statistically less efficient [5]. Further MR studies in larger samples with more cases would be needed for all outcomes. Specially, our results for perinatal depression require further validation using multivariable MR to account for unbalanced horizontal pleiotropy.”

24. Discussion: Please present and organize the Discussion as follows: a short, clear summary of the article's findings; what the study adds to existing research and where and why the results may differ from previous research; strengths and limitations of the study; implications and next steps for research, clinical practice, and/or public policy; one-paragraph conclusion. Specifically, please include a paragraph discussing the implications, next steps for research, clinical practice, and/or public policy between the discussion of study limitations and the overall conclusion paragraph.

Thank you for the suggestion. We have included some relevant information in the conclusion paragraph. Therefore, we have re-organised the last paragraph.

(page 26, line 480) From: “In conclusion, our study raises the possibility of unfavourable associations of insomnia with miscarriage, perinatal depression and LBW. Interventions to improve healthy sleep in women of reproductive age might be beneficial to a health pregnancy. However, we acknowledge the need for further MR studies based on larger GWAS of pregnancy and perinatal outcomes, larger observational studies, and studies in women from ethnic backgrounds other than White European.”

To: “Our findings raise the possibility that interventions to improve healthy sleep (e.g. cognitive behavioural therapy) in women of reproductive age might be beneficial to a health pregnancy. However, we acknowledge the need for further MR studies based on larger GWAS of pregnancy and perinatal outcomes, larger observational studies, and studies in women from ethnic backgrounds other than White European. Further studies on the association of insomnia with recurrent miscarriage would help policy makers decide whether to allocate sleep interventions to women with a history of miscarriage when they prepare to be pregnant again.

In conclusion, our study raises the possibility of associations between insomnia and miscarriage, perinatal depression and LBW.”

25. Discussion: Line 438: We suggest revising to: “In conclusion, our study raises the possibility of associations between insomnia and miscarriage, perinatal depression and LBW.”

We have amended the sentence as suggested.

(page 27, line 488) From: “In conclusion, our study raises the possibility of unfavourable associations of insomnia with miscarriage, perinatal depression and LBW.”

To: “In conclusion, our study raises the possibility of associations between insomnia and miscarriage, perinatal depression and LBW.”

26. Line 443: Data availability statement: Please remove this section from the main text of the manuscript, and make sure all information is completely and accurately entered into the data availability section of the manuscript submission system.

Thank you for the reminder. We have removed data availability statement from the manuscript to the relevant section in the submission system.

27. Line 460: Funding: Please remove this section from the main text of the manuscript, and make sure all information is completely and accurately entered into the funding section of the manuscript submission system.

Thank you for the reminder. We have removed funding statement from the manuscript to the relevant section in the submission system. We have also updated funding information of Born in Bradford as suggested by the cohort.

From: “BiB receives core funding from the Wellcom Trust (WT101597MA), a joint grant from UK MRC and Economic and Social Science Research Councils (MR/N024397/1), BHF (CS/16/4/32482), and NIHR under its Collaboration for Applied Health Research and Care for Yorkshire and Humber and the NIHR Clinical Research Network.”

To: “BiB is supported by a Wellcome programme grant (WT223601/Z/21/Z: Age of Wonder), an infrastructure grant (WT101597MA), a UK MRC and Economic and Social Science Research Council programme grant (MR/N024397/1), a BHF Clinical Study grant (CS/16/4/32482), and NIHR under its Applied Health Research Collaboration Yorkshire and Humber (NIHR200166) and the NIHR Clinical Research Network.”

28. Line 486: Competing interests: Please remove this section from the main text of the manuscript, and make sure all information is completely and accurately entered into the competing interests section of the manuscript submission system.

Thank you for the reminder. We have removed competing interests statement from the manuscript to the relevant section in the submission system. We have also updated Kate Tilling’s statement.

From: “KT has acted as a consultant for CHDI Foundation.”

To: “KT has acted as a consultant for CHDI Foundation, and Expert Witness to the High Court in England, called by the UK Medicines and Healthcare products Regulatory Agency, defendants in a case on hormonal pregnancy tests and congenital anomalies 2021/22.”

29. References: Please use the "Vancouver" style for reference formatting, and see our website for other reference guidelines https://journals.plos.org/plosmedicine/s/submission-guidelines#loc-references

For reference 23, please provide complete information for the reference.

For reference 36, the journal abbreviation should be BMJ.

For reference 37, the journal abbreviation should be JAMA.

For reference 57, the journal abbreviation should be BJOG.

For reference 64, the journal abbreviation should be Nat Rev Methods Primers.

Thank you for pointing these out. We have updated our reference list as suggested. Please see the revised manuscript.

30. Table 1: It might be helpful to clarify in the legend that “miscarriage/stillbirth” represent any history of those (if this is accurate).

We have replaced “Stillbirth” and “Miscarriage” with “History of stillbirth” and “History of miscarriage”, respectively, in the first column of Table 1.

31. Figure 2: Please present the results taking Steiger filtering into account for all of the outcomes, not just those that were significant. Please present this in the figure rather than mentioning the results in the legend. Please present p values in addition to 95% CIs for each association.

Thank you for the suggestion. We have moved IVW results after Steiger filtering into the Figure for stillbirth, perinatal depression and low birthweight. For other outcomes, Steiger filtering did not suggest a removal of any SNPs (with results shown in S3 Table), so there are no additional results.

We have added p-values for each association.

In the legend,

From: “^c^ After Steiger filtering, estimates were 0.83 (0.27, 2.54), 3.57 (1.49, 8.54) and 2.18 (1.13, 4.22) for stillbirth, perinatal depression and LBW, respectively.”

To: “^b^ Steiger filtering did not suggest a removal of any SNPs from MR analyses (details shown in S3 Table).”

32. Figure 3: Please correct the title to: “...insomnia at 18 weeks of gestation…”Please present p values in addition to 95% CIs for each association.

Thank you for pointing this out. We have amended the title and added the p-values into the figure.

From: “Fig 3. Multivariable regression associations of insomnia at 18 of gestation with adverse pregnancy and perinatal outcomes in Avon Longitudinal Study of Parents and Children (ALSPAC)”

To: “Fig 3. Multivariable regression associations of insomnia at 18 weeks of gestation with adverse pregnancy and perinatal outcomes in Avon Longitudinal Study of Parents and Children (ALSPAC)”

33. S3 Table: Please clarify where the footnotes indicating the Steiger filtering of individual SNPs are indicated (e.g. footnotes d, e, f were not found in the table).

Thank you for pointing this out. We have labelled 26 SNPs with “d”, 1 SNP (rs113092074) with “e” and 9 SNPs with “f”. Please see the revised S3 Table.

34. S7 Table: Please move this table to the main text (at least the 18 weeks gestation dataset). Please clarify what is meant by “Insomnia at the other time point” in the variables list.

Thank you for the suggestion. We have moved information related to insomnia 18 weeks of gestation to Table 2 in the main text, and kept information related to insomnia at 32 weeks of gestation into S7 Table.

In Results,

(page 19, line 345) From: “S7 Table summarizes the characteristics of women from ALSPAC who contributed to these analyses.”

To: “Table 2 and S7 Table summarizes the characteristics of women from ALSPAC.”

In Table 2, we clarify “insomnia at the other time point” as “insomnia at 32 weeks of gestation”.

In Table S7, we clarify “insomnia at the other time point” as “insomnia at 18 weeks of gestation”.

From: “S7 Table. Characteristics of women in Avon Longitudinal Study of Parents and Children (ALSPAC) used in confounder-adjusted multivariable regression”

To: “S7 Table. Characteristics of women in Avon Longitudinal Study of Parents and Children by insomnia at 32 weeks of gestation”

35. S6 Fig. Please display all plots on the same axis scale. Please describe interpretations in the figure legend (e.g. attenuation of relationships).

Thank you for the suggestion. We have All plots zoomed out some of them to make all plots have the same axes. Please see the revised S6 Fig.

In legend, we add: “We consider a significant alteration of SNP-outcome associations if the slope is ≤0.8 or ≥1.2.”

Comments from the reviewers:

Reviewer #1: Thank you for the thoughtful responses from the authors. I think the authors have addressed most concerns from me and other reviewers. However, there are still a few remaining concerns I have about the current manuscript.

1. Response to my point # 5:

The explanations given by the authors are great. I think it would be great to include these detailed explanations in the manuscript (possibly in the discussion section). The current revisions made by the authors are insufficient to me.

Thank you for the suggestion. We have added our previous explanations into the main text.

(page 9, line 171) From: “To avoid potential selection bias [6], we randomly selected one pregnancy per woman.”

To: “Given that choosing the first pregnancy could introduce selection towards younger age, lower parity and higher morbidity of HDP [7], we followed EGG consortium convention [8] to randomly select one pregnancy per woman [6].”

2. Response to my point # 6:

(page 27, line 474) We added: "As the sources of bias in our two methods (MR and multivariable regression) differ, where we have consistency between them this increases our confidence in those consistent results being the causal association [17-21]."

I would still be cautious about making any causal inference/claim here based on the consistency in the results.

Thank you for pointing this out. We have amended this sentence as below.

(page 26, line 465) From: “As the sources of bias in our two methods (MR and multivariable regression) differ, where we have consistency between them this increases our confidence in those consistent results being the causal association [9-13].”

To: “As the sources of bias in our two methods (MR and multivariable regression) differ, consistent results between them could strength confidence in the findings even considering different timings of an exposure [9-13]. Our previous study discussed how timings affected the interpretation of MR estimates for insomnia [2].”

3. Response to my point # 7:

Using conventional linear IV method in nonlinear models can lead to substantial bias (https://pubmed.ncbi.nlm.nih.gov/18546544/). Also, as I mentioned, since estimates of the association between SNP and insomnia are just intermediate results for the IV approach, why do we care so much about the interpretation of the intermediate results? Should we prioritize having unbiased estimates of association between insomnia and perinatal outcomes as these associations are more important?

We reconduct (1) split-sample analyses in UKB by generating SNP-insomnia and SNP-outcome associations in logistic regression; (2) MR analyses using SNP-insomnia associations generated in logistic regression by the GWAS [14], and SNP-outcome associations combining FinnGen and three birth cohorts; and (3) meta-analyses of results from (1) and (2). The new results, together with our original results (presented in Fig2 and S6 Table), are shown below. Given both results are in different units, their magnitudes should not be compared directly. By comparing the directions of those point estimates, we think our conclusions for miscarriage, perinatal depression, and low birthweight would not be changed. We have included (1), (2) and (3) as sensitivity analyses, and made the following changes in the manuscript.

In Methods,

(page 11, line 210) We add: “During peer review, we were asked to re-generate SNP-insomnia associations using logistic regression to repeat MR analyses (details in S2 Text).”

In Results,

(page 19, line 340) We add: “After combining all cohorts, most MR estimates based on SNP-insomnia associations from linear (Fig 2) versus logistic (S6 Table b) regression were in the same directions. An association of lifetime susceptibility to insomnia with HBW was observed using IVW (S6 Table b), which previously had a wide 95% CI including the null (Fig 2).”

In Discussion,

(page 24, line 433) We add: “A previous study indicated potential bias when the standard IV approach (see [15]) was used for a nonlinear model [16]. In our study, using linear versus logistic regression to obtain SNP-insomnia associations showed consistent directions of MR estimates. However, magnitudes of MR estimates cannot be compared directly due to their different scales. Further MR studies of binary exposures could use both approaches to explore an association.”

In S2 Text. Two-sample MR based on SNP-insomnia associations from logistic regression,

We add: “During peer review, we were suggested to re-generate SNP-insomnia associations using logistic regression in order to repeat two-sample MR analyses. Therefore, we reconducted: (1) split-sample analyses in UKB by generating SNP-insomnia and SNP-outcome associations in logistic regression; (2) two-sample MR analyses using SNP-insomnia associations generated in logistic regression by the GWAS where we selected our instruments [14], and the pooled SNP-outcome associations combining ALSPAC, BiB, MoBa and FinnGen; and (3) a meta-analysis of MR estimates from (1) and (2) using fixed-effects (with inverse variance weights) for each insomnia-outcome pair. Consistent with a previous MR study of binary exposures [17], our MR estimates were reported as odds ratios per 1 unit higher log-odds of insomnia.”

New SNP-insomnia associations generated by logistic regression in UKB women are added into S4 Table. New MR results are added into S6 Table.

Table. Two-sample MR estimates (Odds ratio, 95% confidence interval) for causal effects of insomnia on adverse pregnancy and perinatal outcomes

| Outcome | Data | Inverse variance weighted | | Weighted median | | MR-Egger | |
| --- | --- | --- | --- | --- | --- | --- | --- |
|  |  | Original ^a^ | New ^b^ | Original ^a^ | New ^b^ | Original ^a^ | New ^b^ |
| Stillbirth | UK Biobank | 1.26 (0.50, 3.15) | 1.05 (0.86, 1.28) | 2.17 (0.57, 8.33) | 1.18 (0.88, 1.58) | 1.63 (0.25, 10.80) | 1.28 (0.83, 1.97) |
|  | BC + FinnGen | 0.55 (1.74×10^-3^,  171.91) | 0.86 (0.47, 1.59) | 0.85 (1.17×10^-4^,  6130.61) | 0.85 (0.35, 2.05) | 3.47 (6.23×10^-6^,  1.93×10^6^) | 0.39 (0.03, 5.36) |
|  | Meta-analysis | 1.23 (0.50, 3.05) | 1.03 (0.85, 1.25) | 2.13 (0.56, 8.03) | 1.14 (0.86, 1.50) | 1.65 (0.25, 10.76) | 1.08 (0.72, 1.63) |
| Miscarriage | UK Biobank | 1.43 (1.01, 2.01) | 1.08 (1.00, 1.16) | 1.31 (0.77, 2.21) | 1.06 (0.95, 1.18) | 1.06 (0.52, 2.16) | 1.01 (0.87, 1.18) |
|  | BC + FinnGen | 2.38 (1.26, 4.50) | 1.10 (1.03, 1.15) | 2.08 (0.75, 5.74) | 1.11 (1.00, 1.23) | 1.67 (0.40, 6.94) | 1.17 (0.89, 1.55) |
|  | Meta-analysis | 1.60 (1.18, 2.17) | 1.09 (1.04, 1.15) | 1.44 (0.90, 2.30) | 1.09 (1.01, 1.17) | 1.16 (0.61, 2.19) | 1.05 (0.91, 1.20) |
| Gestational | UK Biobank | 5.85 (0.57, 60.34) | 1.46 (0.88, 2.43) | 2.19 (0.06, 84.11) | 1.20 (0.54, 2.64) | 0.17 (0.00, 21.01) | 0.67 (0.24, 1.92) |
| diabetes | BC + FinnGen | 0.95 (0.39, 2.33) | 0.98 (0.89, 1.08) | 1.19 (0.30, 4.65) | 0.96 (0.83, 1.11) | 0.99 (0.14, 7.11) | 0.86 (0.58, 1.26) |
|  | Meta-analysis | 1.20 (0.52, 2.77) | 1.00 (0.91, 1.10) | 1.28 (0.36, 4.60) | 0.97 (0.84, 1.11) | 0.77 (0.12, 4.77) | 0.83 (0.58, 1.19) |
| HDP | UK Biobank | 0.63 (0.16, 2.55) | 0.91 (0.67, 1.23) | 0.42 (0.05, 3.60) | 0.82 (0.53, 1.27) | 0.22 (0.00, 15.56) | 0.94 (0.50, 1.76) |
|  | BC + FinnGen | 2.91 (1.05, 8.05) | 1.12 (1.01, 1.25) | 2.14 (0.59, 7.75) | 1.16 (1.02, 1.31) | 1.97 (0.20, 19.41) | 1.15 (0.73, 1.80) |
|  | Meta-analysis | 1.71 (0.75, 3.89) | 1.10 (0.99, 1.21) | 1.39 (0.46, 4.20) | 1.13 (1.00, 1.27) | 1.34 (0.22, 8.04) | 1.07 (0.75, 1.54) |
| Perinatal | UK Biobank | 3.33 (1.25, 8.85) | 1.30 (1.05, 1.61) | 1.36 (0.31, 6.04) | 1.07 (0.78, 1.48) | 0.34 (0.05, 2.54) | 0.80 (0.51, 1.23) |
| depression | BC + FinnGen | 4.68 (0.67, 4.39) | 1.20 (0.98, 1.48) | 1.73 (0.10, 30.80) | 1.27 (0.94, 1.71) | 1.78 (0.02, 161.44) | 1.40 (0.58, 3.38) |
|  | Meta-analysis | 3.56 (1.49, 8.54) | 1.25 (1.08, 1.45) | 1.43 (0.38, 5.38) | 1.17 (0.94, 1.46) | 0.45 (0.07, 2.81) | 0.89 (0.60, 1.31) |
| Preterm  Birth | UK Biobank | 0.12 (0.01, 2.05) | 0.63 (0.34, 1.17) | 0.33 (3.91×10^-3^,  27.22) | 0.76 (0.30, 1.93) | 0.19 (5.45×10^-4^,  62.79) | 0.69 (0.19, 2.44) |
|  | BC + FinnGen | 1.71 (0.67, 4.39) | 1.09 (0.99, 1.21) | 1.18 (0.32, 4.39) | 1.12 (0.98, 1.29) | 0.31 (0.04, 2.44) | 0.95 (0.63, 1.42) |
|  | Meta-analysis | 1.31 (0.54, 3.21) | 1.08 (0.97, 1.19) | 1.06 (0.30, 3.75) | 1.11 (0.97, 1.28) | 0.29 (0.04, 2.05) | 0.92 (0.62, 1.35) |
| Low | UK Biobank | 2.96 (1.59, 5.61) | 1.27 (1.10, 1.46) | 1.56 (0.64, 3.79) | 1.10 (0.91, 1.34) | 1.50 (0.39, 5.72) | 1.09 (0.82, 1.46) |
| birthweight | BC + FinnGen | 9.12 (0.72, 115.53) | 1.31 (1.00, 1.72) | 2.54 (0.05, 121.09) | 1.39 (0.92, 2.10) | 0.42 (1.29×10^-3^,150.74) | 1.02 (0.32, 3.30) |
|  | Meta-analysis | 3.17 (1.69, 5.96) | 1.28 (1.13, 1.44) | 1.60 (0.67, 3.80) | 1.15 (0.97, 1.37) | 1.41 (0.38, 5.20) | 1.09 (0.82, 1.44) |
| High | UK Biobank | 1.38 (0.40, 4.80) | 1.07 (0.82, 1.41) | 0.60 (0.09, 4.04) | 0.90 (0.60, 1.35) | 1.31 (0.10, 17.18) | 1.06 (0.61, 1.86) |
| birthweight | BC + FinnGen | 20.32 (1.82,  226.43) | 1.39 (1.07, 1.79) | 12.09 (0.26,  553.55) | 1.52 (1.04, 2.24) | 25.07 (0.10,  6210.55) | 2.52 (0.85, 7.46) |
|  | Meta-analysis | 2.43 (0.81, 7.34) | 1.23 (1.02, 1.48) | 1.09 (0.20, 6.04) | 1.19 (0.90, 1.57) | 2.22 (0.22, 22.89) | 1.27 (0.77, 2.09) |

^a^ Odds ratio in a risk difference scale (i.e. insomnia versus no insomnia). ^b^ Odds ratio per 1 unit higher log-odds of insomnia [17].

Abbreviations: BC, birth cohorts; HDP, hypertensive disorders of pregnancy.

**Reference:**

1.	Yang Z, Zhu Z, Wang C, Zhang F, Zeng H. Association between adverse perinatal outcomes and sleep disturbances during pregnancy: a systematic review and meta-analysis. J Matern Fetal Neonatal Med. 2020: 1-9.

2.	Yang Q, Sanderson E, Tilling K, Borges MC, Lawlor DA. Exploring and mitigating potential bias when genetic instrumental variables are associated with multiple non-exposure traits in Mendelian randomization. 2022 May 27. doi: <https://doi.org/10.1007/s10654-022-00874-5>.

3.	Lu Q, Zhang X, Wang Y, Li J, Xu Y, Song X, et al. Sleep disturbances during pregnancy and adverse maternal and fetal outcomes: a systematic review and meta-analysis. Sleep Med Rev. 2020;58: 101436.

4.	Cronin RS, Li M, Thompson JMD, Gordon A, Raynes-Greenow CH, Heazell AEP, et al. An Individual Participant Data Meta-analysis of Maternal Going-to-Sleep Position, Interactions with Fetal Vulnerability, and the Risk of Late Stillbirth. EClinicalMedicine. 2019;10: 49-57.

5.	Sanderson E, Glymour MM, Holmes MV, Kang H, Morrison J, Munafò MR, et al. Mendelian randomization. Nat Rev Methods Primers. 2022;2: 6.

6.	Hughes RA, Davies NM, Davey Smith G, Tilling K. Selection Bias When Estimating Average Treatment Effects Using One-sample Instrumental Variable Analysis. Epidemiology. 2019;30: 350-357.

7.	Hernández-Díaz S, Toh S, Cnattingius S. Risk of pre-eclampsia in first and subsequent pregnancies: prospective cohort study. BMJ. 2009;338: b2255.

8.	Beaumont RN, Warrington NM, Cavadino A, Tyrrell J, Nodzenski M, Horikoshi M, et al. Genome-wide association study of offspring birth weight in 86 577 women identifies five novel loci and highlights maternal genetic effects that are independent of fetal genetics. Hum Mol Genet. 2018;27: 742-756.

9.	Lawlor DA, Tilling K, Davey Smith G. Triangulation in aetiological epidemiology. Int J Epidemiol. 2016;45: 1866-1886.

10.	Richmond RC, Timpson NJ, Felix JF, Palmer T, Gaillard R, McMahon G, et al. Using Genetic Variation to Explore the Causal Effect of Maternal Pregnancy Adiposity on Future Offspring Adiposity: A Mendelian Randomisation Study. PLoS Med. 2017;14: e1002221.

11.	Bond TA, Richmond RC, Karhunen V, Cuellar-Partida G, Borges MC, Zuber V, et al. Exploring the causal effect of maternal pregnancy adiposity on offspring adiposity: Mendelian randomisation using polygenic risk scores. BMC Med. 2022;20: 34.

12.	Hernáez Á, Rogne T, Skåra KH, Håberg SE, Page CM, Fraser A, et al. Body mass index and subfertility: multivariable regression and Mendelian randomization analyses in the Norwegian Mother, Father and Child Cohort Study. Hum Reprod. 2021;36: 3141-3151.

13.	Magnus MC, Miliku K, Bauer A, Engel SM, Felix JF, Jaddoe VWV, et al. Vitamin D and risk of pregnancy related hypertensive disorders: mendelian randomisation study. BMJ. 2018;361: k2167.

14.	Jansen PR, Watanabe K, Stringer S, Skene N, Bryois J, Hammerschlag AR, et al. Genome-wide analysis of insomnia in 1,331,010 individuals identifies new risk loci and functional pathways. Nat Genet. 2019;51: 394-403.

15.	Greenland S. An introduction to instrumental variables for epidemiologists. Int J Epidemiol. 2000;29: 722-729.

16.	Terza JV, Bradford WD, Dismuke CE. The use of linear instrumental variables methods in health services research and health economics: a cautionary note. Health Serv Res. 2008;43: 1102--1120.

17.	Østergaard SD, Mukherjee S, Sharp SJ, Proitsi P, Lotta LA, Day F, et al. Associations between Potentially Modifiable Risk Factors and Alzheimer Disease: A Mendelian Randomization Study. PLoS Med. 2015;12: e1001841; discussion e1001841.
