# Supplementary material for: Associations between insomnia and pregnancy and perinatal outcomes: Evidence from mendelian randomization and multivariable regression analyses
Source: PLoS Med. 2022 Sep 6;19(9):e1004090. doi: 10.1371/journal.pmed.1004090 (PMC9488815; doi:10.1371/journal.pmed.1004090)
Supplement: Attachment — Submitted filename: response letter.docx [file pmed.1004090.s018.docx]

Requests from Editors:

1. Abstract: Methods and Findings: Please explicitly state the outcomes of interest early in this section when describing the study methods: ever experiencing stillbirth, ever experiencing miscarriage, GD, HDP, perinatal depression, PTB (gestational age <37 completed weeks), LBW (<2,500 grams) and HBW (>4,500 grams).

Thank you for pointing this out. We have amended the abstract as suggested.

(page 2, line 26) We add: “Our outcomes included ever experiencing stillbirth, ever experiencing miscarriage, gestational diabetes, HDP, perinatal depression, preterm birth (gestational age <37 completed weeks), LBW (<2,500 grams) and HBW (>4,500 grams).

2. Abstract: Methods and Findings: Line 34, Line 41: Here, and throughout, please report p values as p<0.001 where applicable, unless there is a statistical reason to present exact p values smaller than this.

Thank you for the reminder. We have replaced “p-value” with “p” throughout the manuscript. We have also made the following changes. P-values reflecting SNP selection in methods (page 10, line 198) and S3 Table are presented in the exact numbers in order to assure the reproducibility of our analyses.

**In Abstract,**

(page 2, line 36) From: “… LBW (OR 3.17, 95% CI: 1.69, 5.96, p-value=0.0003) …”

To: “… LBW (OR 3.17, 95% CI: 1.69, 5.96, p<0.001) …”

(page 2, line 43) From: “… perinatal depression (OR 2.96, 95% CI: 2.42, 3.63, p-value=2×10^-25^) …”

To: “… perinatal depression (OR 2.96, 95% CI: 2.42, 3.63, p<0.001) …”

**In Results,**

(page 17, line 304) From: “… and 32 (OR 1.02 per effect allele, 95% CI: 1.01, 1.03, p-value=0.0003) … UKB plus 23andMe women (OR 1.05 per effect allele, 95% CI: 1.05, 1.06, p-value=4×10^-271^).”

To: “… and 32 (OR 1.02 per effect allele, 95% CI: 1.01, 1.03, p<0.001) … UKB plus 23andMe women (OR 1.05 per effect allele, 95% CI: 1.05, 1.06, p<0.001).”

(page 20, line 354) From: “… perinatal depression (OR 2.96, 95% CI: 2.42, 3.63, p-value=2×10^-25^, Fig 3).”

To: “… perinatal depression (OR 2.96, 95% CI: 2.42, 3.63, p<0.001, Fig 3).”

**In Fig 2,**

From: “0.0003”

To: “<0.001”

**In Fig 3,**

From: “2E-46”

To: “<0.001”

From: “2E-25”

To: “<0.001”

**In S6 Table,**

From: “3×10^-4^”

To: “<0.001”

From: “2×10^-4^”

To: “<0.001”

3. Abstract: Line 35: Please define “HDP” at first use in the text.

Thank you for pointing this out. We have defined “HDP” in the abstract.

(page 2, line 23) From: “Our aim was to estimate the association of insomnia with stillbirth, miscarriage, gestational diabetes, hypertensive disorders of pregnancy …”

To: “Our aim was to estimate the association of insomnia with stillbirth, miscarriage, gestational diabetes, hypertensive disorders of pregnancy (HDP) …”

4. Abstract: Conclusions: Line 46-47: Please revise to: “In this study, we observed evidence s

We have amended the word as suggested.

(page 3, line 48) From: “In this study, we observed evidence supporting …”

To: “In this study, we observed evidences supporting …”

5. Author summary: Line 59-60: Please revise to: “To the best of our knowledge, Mendelian randomization (MR) has not been used to explore whether there is evidence to support a causal association between insomnia and adverse pregnancy and perinatal outcomes.”

We have amended the sentence as suggested.

(page 4, line 62) From: “To the best of our knowledge, Mendelian randomization (MR) has not been used to explore whether insomnia causes adverse pregnancy and perinatal outcomes.”

To: “To the best of our knowledge, Mendelian randomization (MR) has not been used to explore whether these is evidence to support a causal association between insomnia and adverse pregnancy and perinatal outcomes.”

6. Author summary: We suggest starting off this point to focus on the interpretation of the study findings: “These findings raise the possibility that insomnia may be related to pregnancy outcomes…”.

Thank you for the suggestion. We have made the following change:

(page 4, line 77) From: “These findings raise the possibility that interventions to improve healthy sleep may be beneficial to a healthy pregnancy.”

To: “These findings raise the possibility that insomnia may be related to adverse pregnancy outcomes, implying that interventions to improve healthy sleep may be beneficial to a healthy pregnancy.”

7. Results: Line 294 and 296: Here, and throughout the Results section, please use p<0.001 where appropriate.

We have amended the format of p-values as suggested.

(page 17, line 304) From: “… and 32 (OR 1.02 per effect allele, 95% CI: 1.01, 1.03, p-value=0.0003) … UKB plus 23andMe women (OR 1.05 per effect allele, 95% CI: 1.05, 1.06, p-value=4×10^-271^).”

To: “… and 32 (OR 1.02 per effect allele, 95% CI: 1.01, 1.03, p<0.001) … UKB plus 23andMe women (OR 1.05 per effect allele, 95% CI: 1.05, 1.06, p<0.001).”

(page 20, line 354) From: “… perinatal depression (OR 2.96, 95% CI: 2.42, 3.63, p-value=2×10^-25^, Fig 3).”

To: “… perinatal depression (OR 2.96, 95% CI: 2.42, 3.63, p<0.001, Fig 3).”

8. Results: Line 299-301: Please revise to: “In MR IVW combining all cohorts, point estimates for associations between lifetime susceptibility to insomnia (vs no insomnia) and outcomes ranged from ORs of 1.20 (95% CI: 0.52, 2.77, p value=0.67) for GD, to 3.56 (95% CI: 1.49, 8.54, p-value=0.004) for perinatal depression (Fig 2).”

Thank you for the suggestion. We have rewritten the sentence as suggested.

(page 17, line 309) From: “In MR IVW combining all cohorts, lifetime susceptibility to insomnia (vs no insomnia) was associated with higher risks of all outcomes with point estimate OR ranging from 1.20 (95% CI: 0.52, 2.77, p-value=0.67) for GD, to 3.56 (95% CI: 1.49, 8.54, p-value=0.004) for perinatal depression (Fig 2).”

To: “In MR IVW combining all cohorts, point estimates for associations between lifetime susceptibility to insomnia (vs no insomnia) and outcomes ranged from ORs of 1.20 (95% CI: 0.52, 2.77, p=0.67) for GD, to 3.56 (95% CI: 1.49, 8.54, p=0.004) for perinatal depression (Fig 2).”

9. Results: Line 309: Please revise to: “MR IVW estimates after Steiger filtering were consistent for…”

We have amended the sentence as suggested.

(page 17, line 320) From: “MR IVW estimates were consistent for perinatal depression …”

To: “MR IVW estimates after Steiger filtering were consistent for perinatal depression …”

10. Discussion: Line 362: Please revise to “...provide some evidence that a lifetime susceptibility to insomnia might be associated with….”

We have amended the sentence as suggested.

(page 23, line 376) From: “MR results provide evidence that a lifetime susceptibility to insomnia might associate with higher risks of miscarriage, perinatal depression, and LBW.”

To: “MR results provide some evidence that a lifetime susceptibility to insomnia might be associated with higher risks of miscarriage, perinatal depression and LBW.”

11. Discussion: Line 405-406: Please revise to: “Results from these sensitivity analyses were broadly consistent with point estimates from IVW, however, the associations between insomnia and miscarriage, perinatal depression, and low offspring birth weight no longer reached statistical significance. The wider 95% CIs observed could be attributable to the fact that these sensitivity analyses are known to be statistically less efficient [45].” It might be helpful to provide a sentence of explanation of what is meant by statistically less efficient, and alternative interpretations.

Thank you for the suggestions. Being statistically less efficient means having less power. The alternative interpretation would be weak instrument bias, which is expected to bias point estimates towards the null in two-sample Mendelian randomization analyses [1,2]. We have made the following changes.

(page 25, line 420) From: “Results from these sensitivity analyses were broadly consistent with point estimates from IVW, but with wider 95% CIs given they were known to be statistically less efficient [3].”

To: “Results from these sensitivity analyses were broadly consistent with point estimates from IVW, however, the associations between insomnia and miscarriage, perinatal depression, and LBW no longer reached statistical significance. The wider 95% CIs observed could be attribute to the fact that these sensitivity analyses are known to have less statistical power [2]. Those attenuations towards the null could be due to weak IVs, and MR-Egger point estimates are known to be attenuated more severely than weighted median ones [1,2].”

12. Discussion: Line 464: We suggest revising the first sentence to more closely address the implications of the study without overreaching what can be concluded (i.e. the study does not exactly provide evidence that suggests a sleep intervention could be beneficial in healthy pregnancy). We suggest: “Our findings provide some evidence for associations between insomnia and pregnancy outcomes, raising the possibility that interventions to improve healthy sleep (e.g. cognitive behavioural therapy) in women of reproductive age might be beneficial strategy to promote healthy pregnancy.” or similar.

Thank you very much for the suggestion. We have amended the sentence accordingly.

(page 27, line 482) From: “Our findings raise the possibility that interventions to improve healthy sleep (e.g. cognitive behavioural therapy) in women of reproductive age might be beneficial to a healthy pregnancy.”

To: “Our findings provide some evidence for associations between insomnia and adverse pregnancy outcomes, raising the possibility that interventions to improve healthy sleep (e.g. cognitive behavioural therapy) in women of reproductive age might be beneficial to a healthy pregnancy.”

13. References: When providing a DOI please include both the label and full DOI included at the end of the reference (e.g. doi: 10.1016/j.molimm.2014.11.005). Please do not provide a shortened DOI or the URL.

Thank you for pointing this out. We have updated the reference list.

(page 31, line 563) From: “Yang Q, Sanderson E, Tilling K, Borges MC, Lawlor DA. Exploring and mitigating potential bias when genetic instrumental variables are associated with multiple non-exposure traits in Mendelian randomization. 2022 May 27. doi: <https://doi.org/10.1007/s10654-022-00874-5>.”

To: “Yang Q, Sanderson E, Tilling K, Borges MC, Lawlor DA. Exploring and mitigating potential bias when genetic instrumental variables are associated with multiple non-exposure traits in Mendelian randomization. 2022;37: 683-700.”

14. Figure 3: Please use p<0.001 for p values when appropriate (for perinatal depression).

Thank you for the reminder. We have replaced “0.0003” in Figure 2, and “2E-46” and “2E-25” in Figure 3 all with “<0.001”. Please see the revised figures.

15. S6 Table: Please provide p values for associations reported.

Thank you for the reminder. We have added p-values for the associations in S6 Table and S2 Fig. Please see the revised supplementary files.

16. Table 1, Table 2, and S7 Table: Please define the abbreviations used for maternal education (GCSE) in the legend.

Thank you for pointing this out. We have added the following legend under Table 1, Table 2 and S7 Table.

(page 18, line 328 & page 21, line 359) We add: “O level, General Certificate Education (GCE) Ordinary Level; GCSE, General Certificate of Secondary Education; A level, GCE Advanced level; AS level, GCE Advanced Subsidiary level.”

17. S2 Text: We suggest moving this to the main text of the Methods section at line 206.

Thank you for the suggestion. We have moved S2 Text into Methods.

(page 11, line 208) From: “During peer review, we were asked to re-generate SNP-insomnia associations using logistic regression to repeat MR analyses (details in S2 Text).”

To: “During peer review, we were suggested to re-generate SNP-insomnia associations using logistic regression to repeat two-sample MR analyses. Therefore, we reconducted: (1) split-sample analyses in UKB by generating SNP-insomnia and SNP-outcome associations in logistic regression; (2) two-sample MR using SNP-insomnia associations generated in logistic regression by the GWAS where we selected our IVs [4], and the pooled SNP-outcome associations combining ALSPAC, BiB, MoBa and FinnGen; and (3) a meta-analysis of MR estimates from (1) and (2) using fixed-effects (with inverse variance weights) for each insomnia-outcome pair. Consistent with a previous MR study of binary exposures [5], our MR estimates were reported as odds ratios per 1 unit higher log-odds of insomnia.”

**References:**

1.	Lawlor DA. Commentary: Two-sample Mendelian randomization: opportunities and challenges. Int J Epidemiol. 2016;45:908-15.

2.	Burgess S, Foley CN, Allara E, Staley JR, Howson JMM. A robust and efficient method for Mendelian randomization with hundreds of genetic variants. Nat Commun. 2020;11:376.

3.	Sanderson E, Glymour MM, Holmes MV, Kang H, Morrison J, Munafò MR, et al. Mendelian randomization. Nat Rev Methods Primers. 2022;2:6.

4.	Jansen PR, Watanabe K, Stringer S, Skene N, Bryois J, Hammerschlag AR, et al. Genome-wide analysis of insomnia in 1,331,010 individuals identifies new risk loci and functional pathways. Nat Genet. 2019;51:394-403.

5.	Østergaard SD, Mukherjee S, Sharp SJ, Proitsi P, Lotta LA, Day F, et al. Associations between Potentially Modifiable Risk Factors and Alzheimer Disease: A Mendelian Randomization Study. PLoS Med. 2015;12:e1001841.
